# A vast stem‐progenitor cell pool, richly vascular system, and hybrid ossification drive the daily centimeter‐scale elongation of bony antlers

**DOI:** 10.1002/imt2.70097

**Published:** 2025-12-08

**Authors:** Hengxing Ba, Shidian He, Hai‐Xi Sun, Xin Wang, Hang Zhang, Qiuting Deng, Yue Yuan, Chang Liu, Zhen Wang, Jiping Li, Liuwei Xie, Yujiao Tang, Jimei Wang, Chao Ma, Nan Li, Pengfei Hu, Qianqian Guo, Guokun Zhang, Dawn Elizabeth Coates, Ying Gu, Chuanyu Liu, Datao Wang, Chunyi Li

**Affiliations:** ^1^ Jilin Provincial Key Laboratory of Deer Antler Biology, Institute of Antler Science and Product Technology Changchun Sci‐Tech University Changchun China; ^2^ College of Life Sciences Jilin Agricultural University Changchun China; ^3^ BGI Research Beijing China; ^4^ College of Life Sciences University of Chinese Academy of Sciences Beijing China; ^5^ State Key Laboratory of Genome and Multi‐omics Technologies, BGI Research Shenzhen China; ^6^ BGI Shenzhen China; ^7^ BGI Cell Shenzhen China; ^8^ Institute of Special Economic Animals and Plants, Chinese Academy of Agricultural Sciences Changchun China; ^9^ State Key Laboratory of Genome and Multi‐omics Technologies, BGI Research Hangzhou China; ^10^ Shenzhen Proof‐of‐Concept Center of Digital Cytopathology, BGI Research Shenzhen China; ^11^ Department of Police Dog Technology Criminal Investigation Police University of China Shenyang China; ^12^ Department of Stomatology Shenzhen People's Hospital (Second Clinical Medical School of Jinan University; First Affiliated Hospital of Southern University of Science and Technology) Shenzhen China; ^13^ Sir John Walsh Research Institute, Faculty of Dentistry University of Otago Dunedin New Zealand

## Abstract

Bone growth and regeneration remain major clinical challenges. Deer antlers, the fastest‐growing mammalian bone, regenerate via endochondral ossification and elongate up to 2 cm per day, far surpassing the ~2 cm annual growth of human growth plates. Here, we systematically mapped the cellular landscape of the antler growth center (AGC) using single‐nucleus RNA sequencing, chromatin accessibility profiling, and spatial transcriptomics. The AGC harbors a large stem‐progenitor pool that drives rapid elongation through vigorous proliferation supported by paracrine signaling. These proliferative cells exhibit a transcriptional program with intrinsically low tumorigenic potential, associated with apoptotic regulation. The AGC also establishes a vascularized niche that supports robust angiogenesis, sustains accelerated cartilage growth, and enables efficient recruitment of osteogenic cells. Notably, antlers employ a hybrid ossification strategy, combining endochondral ossification with direct hypertrophic chondrocyte‐to‐osteoblast transdifferentiation, likely via *PHEX*⁺ intermediates. Collectively, these findings refine fundamental concepts of endochondral ossification and offer insights for regenerative bone therapies.

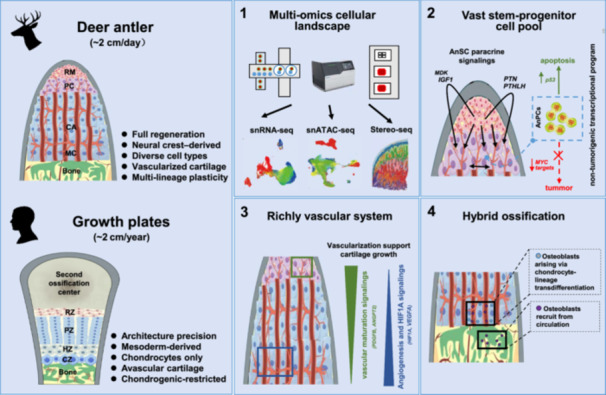

To the editor,

Deer antlers can elongate up to ~2 cm per day, over 300 times faster than the ~2 cm annual extension of human growth plates, representing the fastest bone regeneration in mammals [1, 2]. Such rapid regrowth defies conventional paradigms of endochondral ossification, which typically depend on avascular growth plates and slow chondrocyte maturation. Instead, antlers regenerate through a vascularized cartilage matrix that continuously transitions into bone. This process is orchestrated by the antler growth center (AGC), a highly vascularized niche that supports rapid endochondral ossification [3]. Antler mesenchymal stem cells (AnSCs), derived from cranial neural crest cells [4], are central to this mechanism, yet how their progeny achieve fast and orderly elongation remains unclear. Understanding this unique system provides mechanistic insights into an alternative mode of skeletal growth and may guide the development of strategies that promote bone formation and regeneration while minimizing oncogenic risk.

Here, using an integrated single‐cell multi‐omics approach, we identify three key innovations that underpin the extraordinary elongation of antlers: extensive progenitor proliferation with inherently low tumorigenic potential, vascularized cartilage that meets intense metabolic demands, and a hybrid mode of ossification. Together, these innovations establish a ‘continuous‐flow’ growth model that enables antlers to sustain daily, centimeter‐scale bone elongation.

## MULTI‐OMICS ATLAS OF THE AGC CELLULAR LANDSCAPE

**Figure 1:**
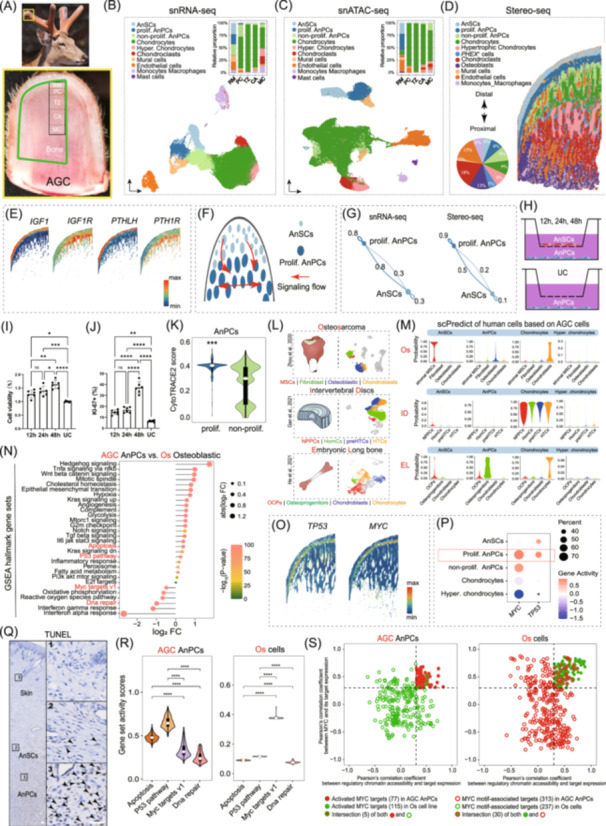
Multi‐omics cellular profiling of the AGC and AnSC‐mediated AnPC proliferation that demonstrate transcriptomic programs distinct from osteosarcoma. (A) Schematic illustration showing the anatomical location of the AGC and sampling sites for snRNA‐seq, snATAC‐seq, and Stereo‐seq. The AGC comprises five histologically distinguishable layers arranged distoproximally: reserve mesenchyme (RM), pre‐cartilage (PC), transition zone (TZ), cartilage (CA), and mineralized cartilage (MC). Note that samples did not include the dermal region and Stereo‐seq included proximal bone tissue. Tissues for the snRNA‐seq were collected from three healthy 2‐year‐old sika deer approximately 30 days of growth after casting of their previous hard antlers. The distal 8 cm portion of the AGC was excised and sectioned sagittally along the longitudinal axis. Five distinguishable tissue layers of the AGC were immediately identified, dissected, and processed as previously described. For the Stereo‐seq, a relevant area of 1 cm × 2 cm was selected, which is the maximum size that can be accommodated by the technique. To fit this size, a first growing antler (approximately 10 days old) from a healthy 1‐year‐old sika deer was used, allowing the 1 cm × 2 cm section to encompass all five tissue layers along with the underlying bone tissue. (B) UMAP plot of the snRNA‐seq data showing 10 distinct cell types, along with a bar plot displaying the relative proportions of each cell type across the five tissue layers. (C) UMAP plot of the snATAC‐seq data showing consistent cell type identities, accompanied by a bar plot illustrating their relative proportions across the five tissue layers. (D) Spatial map showing the distribution of 11 cell types. Notably, two cell types, *PHEX* ^+^ cells and osteoblasts, were identified in this Stereo‐seq analysis, while mast cells were absent, likely due to their low abundance. The pie chart shows the percentage of each cell type. (E) Spatial expression patterns of *IGF1* and its receptor *IGF1R*, as well as *PTHLH* and its receptor *PTH1R*, in AnSCs and AnPCs. (F) Schematic diagram illustrating the CellChat‐inferred cell‐cell communications between AnSCs and proliferative AnPCs, as well as among proliferative AnPCs. (G) Circle plots showing that the interaction strengths are greater from AnSCs to proliferative AnPCs rather than from proliferative AnPCs to AnSCs, based on snRNA‐seq (left) and Stereo‐seq (right). (H) Schematic of the coculture experiment of AnSCs with AnPCs. UC: untreated control. (I) AnPC viability at 12, 24, and 48 h, showing time‐dependent increase (*n* = 6). (J) AnPC proliferation at 12, 24, and 48 h, showing time‐dependent increase (*n* = 6). Significant differences were determined using a one‐way analysis of variance, followed by Tukey's post hoc test. ****p < 0.0001, **p < 0.01, *p < 0.05. Error bars: SD. (K) Violin plots of CytoTRACE2 scores for proliferative AnPCs and non‐proliferative AnPCs. The Wilcoxon Rank Sum test was performed between the proliferative and non‐proliferative AnPCs: ***p < 0.001. (L) Schematic of analyzed tissues: osteosarcoma (Os; Zhou et al., 2020), intervertebral disc (ID; Gan et al., 2021) and embryonic long bone (EL; He et al., 2021), with UMAP plots showing cartilage/bone‐related cell types. (M) Violin plots showing scPred‐predicted probabilities of cartilage‐ and bone‐related cell types in Os, ID, and EL using AnSC‐derived cells as the reference. (N) Lollipop plot illustrating significant differences in AUCell scores of GSEA hallmark gene sets between AGC AnPCs and Os osteoblasts. Several key hallmark gene sets were highlighted. Significant differences were determined using the Kruskal–Wallis test, followed by post hoc Dunn's test. (O) Spatial expression patterns of *TP53* and *MYC* in AGC, with predominant expression observed in AnPCs. (P) Dot plot displaying chromatin accessibility at the *TP53* and *MYC* locus from snATAC‐seq, with predominant accessibility observed in AnPCs. (Q) TUNEL staining of the antler skin and RM layers (AnSCs and AnPCs). Black arrowheads indicate TUNEL‐positive cells. Apoptotic cells were rarely observed in the skin, but were abundant in AnPCs. Scale bar: 50 μm. (R) Violin plots showing the activity scores of key GSEA hallmark gene sets in AGC AnPCs and Os cells (Pontius et al., 2023), as determined by snATAC‐seq data. Significant differences were determined using the Kruskal–Wallis test, followed by post hoc Dunn's test. ****p < 0.0001. (S) Scatter plot showing the Pearson's correlation coefficient between *MYC* expression and its target gene expression, as well as the coefficient between target gene chromatin accessibility and their gene expression, in AGC AnPCs and Os cells. Notably, only five activated *MYC* target genes in the Os cells were active in AnPCs. In contrast, 30 activated *MYC* target genes in AnPCs were active in Os cells.

We sampled five anatomically defined layers of the AGC: reserve mesenchyme (RM), pre‐cartilage (PC), transition zone (TZ), cartilage (CA), and mineralized cartilage (MC) (Figure 1A), providing a spatially resolved framework of antler elongation. Single‐nucleus RNA‐seq profiled 53,949 high‐quality nuclei (Figure S1A–D) and identified ten cell types (Figure 1B) based on canonical markers [5, 6], including *NT5E* ^+^ *TWIST2* ^+^ *SFRP2* ^+^ *RXFP2* ^+^ AnSCs, *TNC* ^+^ *TNN* ^+^ AnPCs with MKI67^+^ proliferative subsets, *COL2A1* ^+^ chondrocytes, *COL10A1* ^+^ hypertrophic chondrocytes, *ACP5* ^+^ chondroclasts, *ACTA2* ^+^ mural cells, *PECAM1* ^+^ *CDH5* ^+^ endothelial cells, *CSF1R* ^+^ monocytes/macrophages, and *TPSB2* ^+^ mast cells (Figure S2A–C). Proliferative AnPCs constituted a major fraction, underscoring their role in driving rapid growth. Cell‐type‐specific DEGs supported the annotations (Figure S2D). Deconvolution of bulk RNA‐seq data [7] showed congruent cellular compositions (Figure S2E).

Single‐nucleus ATAC‐seq (57,722 cells) recovered the same ten populations (Figure 1C, Figures S3–S4A–C). Cross‐modality integration showed high concordance: 87.6% cell‐type agreement and 98.9% tissue‐layer correspondence, with strong correlations between transcriptomic and chromatin accessibility profiles (Pearson *r* = 0.89–0.99, Figure S4D,E).

Stereo‐seq (57,311 cells) resolved 11 cell populations (Figure 1D, Figures S5–S6), recovering all cell types except mast cells, likely due to their low abundance. Spatial mapping delineated a clear differentiation hierarchy from distal AnSCs to AnPCs, chondrocytes, hypertrophic chondrocytes, and finally *PHEX* ^+^ cells. *PHEX* encodes a metalloproteinase essential for phosphate metabolism and mineralization [8]. *PHEX* ^+^ cells (~5%) were positioned adjacent to hypertrophic chondrocytes and likely represent a terminal chondrogenic state contributing to mineral deposition. Osteoblasts (~13%) were located below the MC layer within the ossification zone, consistent with rapid bone formation.

## ANSC SIGNALING DRIVES ANPC PROLIFERATION, WHICH MAY POSSESS MULTI‐LINEAGE DIFFERENTIATION POTENTIAL

AnPCs contained a distal proliferative subset that supported rapid appositional expansion of antler tissue. *IGF1*, previously shown to stimulate antler cell proliferation in vitro [9], was expressed in AnSCs, while its receptor *IGF1R* was enriched in both proliferative and non‐proliferative AnPCs (Figure 1E). *PTHLH* and its receptor *PTH1R* were also highly expressed in AnSCs and non‐proliferative AnPCs, consistent with their role in delaying differentiation [10]. These patterns suggest that AnSCs maintain AnPC proliferation and prevent premature differentiation through paracrine signaling (Figure 1F).

Cell–cell communication inferred from snRNA‐seq and Stereo‐seq showed stronger outgoing signals from AnSCs toward proliferative AnPCs than vice versa (Figure 1G, Figure S7A–B), supporting their function in sustaining the proliferative progenitor pool. In vitro coculture (Figure 1H) further confirmed that AnSC‐derived factors increased AnPC viability (Figure 1I) and proliferation (Figure 1J), highlighting the central role of AnSC signaling in antler growth.

Proliferative AnPCs were regulated by core transcription factors including *TWIST2*, *MEOX2*, *EGR2*, *PRRX2*, and *ZNF37A* (Figure S7C–E). Enrichment analyses indicated that these cells possess high cellular plasticity and multi‐lineage potential (Figure 1K, Figure S7F–G).

In summary, a large and actively maintained stem‐progenitor pool underlies the centimeter‐scale daily elongation of bony antlers. Unlike somatic growth plates, the AGC operates as a continuous system optimized for maximal elongation. This “continuous‐flow” architecture is orchestrated by systemic endocrine cues [10] and localized autocrine/paracrine growth factor signaling.

## AGC EXHIBITS DISTINCT TRANSCRIPTOMIC PROGRAMS FROM OSTEOSARCOMA DESPITE RAPID GROWTH

Comparative analysis of AGC cells with osteosarcoma (Os) [11], normal intervertebral discs (ID) [12], and embryonic long bones (EL) [13] revealed that AGC AnSCs transcriptionally resembled stromal mesenchymal stem cells from Os but represented normal, nonmalignant populations. Despite their high proliferative activity, AnPCs showed minimal similarity to Os osteoblasts, which correspond to malignant populations (Figure 1L,M, Figure S8A–E). GSEA hallmark analysis further distinguished AGC AnPCs from Os osteoblasts: apoptosis‐ and p53‐related pathways were enriched in AnPCs, whereas DNA repair and *MYC* target gene sets predominated in Os osteoblasts (Figure 1N). Apoptosis‐related genes (*TP53*, *CASP3*, *ANXA5*, *ANXA6*, *BAK1*, and *BID*) were highly expressed (Figure 1O, Figure S9A–B), and *TP53* exhibited increased chromatin accessibility in AnPCs (Figure 1P), consistent with substantial apoptosis confirmed by TUNEL staining in the proliferative zone (Figure 1Q). Although MYC was highly expressed (Figure 1O) and chromatin‐accessible (Figure 1P) in AnPCs, snATAC‐seq revealed markedly lower activity of *MYC* target genes compared with Os cells [14] (Figure 1R). Only five activated *MYC* targets in Os cells were shared with AnPCs, whereas AnPCs activated 30 *MYC* targets present in Os cells (Figure 1S). Collectively, these results indicate that rapid antler elongation is maintained by a controlled balance of proliferation and apoptosis [15] rather than oncogenic programs or enhanced DNA repair. In AnPCs, *MYC* drives an alternative, non‐tumorigenic transcriptional program that supports fast growth while avoiding malignant transformation.

## THE VASCULARIZED AGC MICROENVIRONMENT SUPPORTS VASCULAR DEVELOPMENT, FACILITATING CARTILAGE GROWTH AND OSSIFICATION

**Figure 2:**
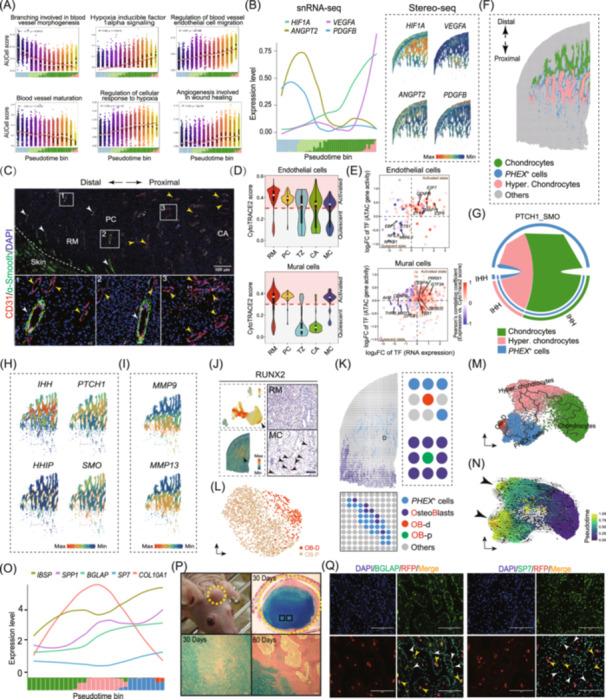
AnSC‐derived cells create a vascularized niche that drives vascular development. *PHEX*⁺ cells promote cartilage mineralization via Hedgehog signaling and may serve as intermediates in the direct conversion of hypertrophic chondrocytes to osteoblasts. (A) Scatter plot showing AUCell scores for vascular development‐related biological process (BP) signaling pathways along AnSC‐derived cell trajectories. The proportion of AnSC‐derived cells along pseudotime is shown in bins (bottom). Colored cell types are indicated in Figure 1B. (B) Expression dynamics of representative marker genes from the snRNA‐seq (left) and the Stereo‐seq (right) along the pseudotime bin. (C) Immunofluorescence staining of the AGC reveals vascular development. Vessels composed only of endothelial cells (yellow arrowheads) indicate angiogenesis, whereas mature vessels are marked by white arrowheads. Red, endothelial cells; green, mural cells. (D) Violin plots of CytoTRACE2 scores for endothelial and mural cells across five tissue layers. Activated and quiescent states were defined using a CytoTRACE2 threshold of 0.3. (E) Scatter plots of transcription factors (TFs) correlated with activated or quiescent states. Pearson's correlation coefficients between TF expression and their CytoTRACE2 scores are shown; the top five TFs are labeled. (F) Spatial distribution of chondrocytes, hypertrophic chondrocytes, and *PHEX* ^+^ cells. (G) Circle plot showing CellChat‐inferred Hedgehog (HH) pathway interactions among these cell types. (H) Spatial expression patterns of representative HH signaling genes. (I) Spatial expression patterns of *MMP9* and *MMP13* genes. (J) UMAP plot (upper left) and spatial map (lower left) showing *RUNX2* expression. Immunohistochemical staining confirms *RUNX2* protein expression in the RM (upper right) and MC (lower right) layers. Notably, the RM layer contains a substantial number of positive cells. In the MC layer, positive hypertrophic chondrocytes are indicated by black arrowheads. Scale bar, 50 μm. (K) Spatial localization of *PHEX* ^+^ cells and osteoblasts. Cell type localization (upper left) and enlarged region of interest (lower left). Osteoblasts are divided into distal (OB‐p, green) and proximal (OB‐d, red) populations relative to *PHEX* ^+^ cells, as illustrated (right). (L) UMAP plot showing distinct clustering of OB‐d and OB‐p populations. (M) Monocle3‐inferred cellular trajectory embedded in UMAP space, showing developmental progression from chondrocytes and hypertrophic chondrocytes through *PHEX* ^+^ cells toward OB‐d. OB‐d cells form a distinct terminal cluster. (N) StaVia‐inferred pseudotime trajectory embedded in the same UMAP space, illustrating differentiation flow from chondrocytes and hypertrophic chondrocytes through *PHEX* ^+^ cells toward OB‐d. Two trajectory endpoints are evident: hypertrophic chondrocytes and OB‐d (black arrowheads). (O) Expression dynamics of representative marker genes from the snRNA‐seq along the pseudotime bin. Colored cell types refer to those shown in (M). (P) Xenogeneic antler formation model showing avascular cartilage (AC‐antler). H&E and Alcian blue staining at Day 30 revealed a nodule of avascular cartilage with early ossification (black boxes), which expanded by Day 60. (Q) Immunofluorescence analysis of the xenogeneic vascularized cartilage model (VC‐antler, day 60) using RFP‐expressing nude mice. *BGLAP* (cytoplasmic) and *SP7* (nuclear) expression are shown. Red fluorescent (RFP^+^) host cells primarily contributed to vascular formation. Osteoblasts derived from perivascular bone marrow–derived mesenchymal stem cells are indicated by yellow arrowheads (OB‐p), while those transdifferentiated from hypertrophic chondrocytes/*PHEX* ^+^ cells are marked with white arrowheads (OB‐d). Not all positive cells are labeled. Scale bar, 200 μm.

To investigate how the AGC microenvironment supports vascular development, we analyzed AUCell activities of vascular‐related pathways along the AnSC lineage trajectory (Figure 2A, Figure S10). The proximal AGC exhibited a hypoxic niche, with strong enrichment of *HIF1A* signaling (*r* = 0.84), cellular response to hypoxia (*r* = 0.89), endothelial migration (*r* = 0.84), and angiogenesis (*r* = 0.84), accompanied by high expression of *HIF1A* and *VEGFA* (Figure 2B). In contrast, the distal AGC was enriched for vessel branching (*r* = 0.77) and vascular maturation (*r* = 0.82), with elevated *PDGFB* and *ANGPT1* expression (Figure 2B). Immunofluorescence staining confirmed findings of these spatial patterns (Figure 2C).

Mural and endothelial cells were predominantly mitotically active within the RM and PC layers (Figure S11A,B), with a significantly higher proportion of activated cells (CytoTRACE2 > 0.3; Figure 2D). Cross‐tissue comparisons showed that activated mural and endothelial cells were more abundant in the AGC than in ID or Os (Figure S11C). These cells exhibited a proangiogenic profile, upregulating *PTN‐SDC1*, *MDK‐SDC1*, *WNT5A‐FZD*, and *VEGFA‐KDR*, while pathways linked to vascular maturation or calcification (*BMP2/4/6‐BMPR1A/BMPR2*, *VEGFA‐FLT1*, and *PDGFA/D‐PDGFRB*) were downregulated (Figure S11D,E). Transcription factor activity positively correlated with differentiation potential (Figure 2E), highlighting a regenerative vascular network optimized for active angiogenesis rather than terminal stabilization.

Compared with avascular somatic growth plates, vascularized cartilage in the AGC confers three advantages: (1) enhanced metabolic support, as hypertrophic chondrocytes maintain direct vascular contact and avoid hypoxia‐induced apoptosis; (2) spatial flexibility, enabling rapid appositional cartilage addition; and (3) efficient ossification, as osteoblasts can immediately access mineralization fronts via vessels. These innovations overcome the metabolic limits that typically constrain hypertrophic expansion in avascular cartilage.

## *PHEX* ^+^ CELLS CONTRIBUTE TO ANTLER CARTILAGE MINERALIZATION VIA HEDGEHOG SIGNALING

*PHEX* ^+^ cells represent ~5% of total cell populations, spatial mapping showed they localize mainly around hypertrophic chondrocytes (Figure 2F). Both snRNA‐seq (Figure S12A–C) and snATAC‐seq (Figure S12D–F) confirmed that *PHEX* ^+^ cells are a chondrocyte subset. Their highly expressed genes were significantly enriched in bone mineralization (*p* = 8.16 × 10⁻⁷), including *LGR4*, *FITM5*, *ASPN*, *IBSP*, *LOX*, and *BGLAP* (Figure S12G,H). CellChat analysis revealed strong signaling from chondrocytes and hypertrophic chondrocytes to *PHEX* ^+^ cells, with Hedgehog (HH) signaling as a key axis (Figure 2G, Figure S12I). IHH was expressed in chondrocytes, while *PTCH1* and *SMO* were enriched in *PHEX* ^+^ cells (Figure 2H), indicating ligand‐receptor crosstalk. *HHIP*, an inhibitory feedback factor, was also elevated in *PHEX* ^+^ cells, suggesting fine‐tuning of HH signaling for mineralization homeostasis. Additionally, *MMP9* and *MMP13* expression (Figure 2I) may promote vascular invasion and osteoblast recruitment, facilitating bone matrix deposition.

## *PHEX* ^+^ CELLS MAY SERVE AS INTERMEDIATES IN HYPERTROPHIC CHONDROCYTE‐TO‐OSTEOBLAST TRANSDIFFERENTIATION

Enrichment analysis revealed that genes highly expressed in *PHEX* ^+^ cells (e.g., *IBSP*, *BGLAP*, *SPP1*, *SP7*, *LOX*, and *COL1A1*; Figure S12G,H,J) are involved in osteoblast differentiation (*p* = 6.83 × 10⁻¹³), indicating an osteoblast‐like transcriptional program. During endochondral ossification, a subset of hypertrophic chondrocytes can evade apoptosis and transdifferentiate into osteoblasts, a process facilitated by *RUNX2* [16]. In the AGC, *RUNX2* was strongly expressed in both AnPCs and hypertrophic chondrocytes (Figure 2J), suggesting that hypertrophic chondrocytes may give rise to osteoblasts via *PHEX* ^+^ intermediates. However, direct lineage tracing in deer is currently unfeasible.

To address this limitation, we leveraged the spatially resolved architecture of the AGC and identified two osteoblast subtypes: OB‐d, which were located exclusively adjacent to *PHEX* ^+^ cells; and OB‐p, which were situated in regions lacking direct contact with *PHEX* ^+^ cells (Figure 2K). Dimension reduction analysis confirmed that OB‐d and OB‐p represent two distinct osteoblast populations (Figure 2L, Figure S12K). Furthermore, differentiation flow along the pseudotime trajectory illustrated a developmental progression from chondrocytes and hypertrophic chondrocytes, through *PHEX* ^+^ cells, toward OB‐d cells (Figure 2M). Notably, two trajectory endpoints were observed: one at hypertrophic chondrocytes and the other at OB‐d cells (Figure 2N). This shows that a small subset of hypertrophic chondrocytes transdifferentiated into OB‐d cells via a *PHEX* ^+^ intermediate. Along this pseudotime trajectory, osteoblast‐related genes (e.g., *IBSP*, *SPP1*, *BGLAP*, and *SP7*) were progressively upregulated, while the hypertrophic chondrocyte marker *COL10A1* was downregulated during the transdifferentiation process (Figure 2O).

To further validate this transdifferentiation in vivo, we used a xenogeneic antler transplantation model in nude mice. Avascularized cartilage nodules were generated via transplantation of antler stem cell tissue (AP) [17]. By Day 30 post‐transplantation, osteoblasts began to appear at the center of the large avascularized cartilage nodules, without evidence of chondroclasia. By Day 60, osteoblasts were broadly distributed within trabeculae and woven bone (Figure 2P). In parallel, vascularized cartilage was generated in RFP (red fluorescent protein)‐expressing nude mice, with the skull periosteum thoroughly scraped at the transplantation site. Previous studies have shown that vascular endothelial cells in these xenogeneic antlers are predominantly derived from the RFP^+^ host mice [18]. Immunofluorescence analysis of osteoblast markers (*BGLAP* and *SP7*) revealed two distinct regions of expression: one surrounding blood vessels (OB‐p), representing osteoblasts differentiated from perivascular bone marrow mesenchymal stem cells, and another within the avascularized cartilage/bone nodules (OB‐d), consistent with osteoblasts transdifferentiated from chondrocytes (Figure 2Q). Although direct lineage tracing is not feasible in cervids, this transplantation model provides the strongest attainable in vivo validation. It clearly demonstrates chondrocyte‐to‐osteoblast transition, even though it does not directly capture the rare *PHEX* ^+^ intermediates. Notably, our observations align with well‐established principles in mammalian bone biology, in which terminal hypertrophic chondrocytes give rise to osteoblasts during embryonic and early postnatal endochondral ossification [19]. Importantly, the osteoblasts derived from the peripheral bone marrow lineage (OB‐p) are the primary contributors to rapid antler ossification [20]. Collectively, these results provide compelling evidence that a small subset of osteoblasts within the AGC originates via direct transdifferentiation from chondrocyte‐lineage cells, with *PHEX* ^+^ cells potentially serving as critical intermediate states.

Antlers achieve unparalleled rapid elongation by expanding proliferative zones both spatially and temporally, with minimal mechanical load at the tip. In contrast, somatic growth plates prioritize structural precision and strength to support body weight and locomotion. Antlers vascularize early to meet extreme metabolic demands, whereas growth plates remain avascular to preserve zonal organization. During ossification, antlers convert apoptotic signals into cues for chondrocyte transdifferentiation, while growth plates enforce terminal differentiation. Growth plates thus function like compartmentalized assembly lines, whereas antlers act as “continuous‐flow” systems that minimize transitional delays, enabling exceptional growth. These adaptations likely evolved under strong selection for rapid seasonal regeneration, allowing large species such as wapiti to grow antlers up to ~1.5 m and ~30 kg within ~90 days [2, 20]. Future work should explore whether these mechanisms can be applied to human bone tissues that require rapid regeneration or repair in the clinical setting.

## AUTHOR CONTRIBUTIONS

**Hengxing Ba**: Conceptualization; formal analysis; supervision; writing—review and editing; writing—original draft; visualization; project administration; validation. **Shidian He**: Writing—original draft; software; formal analysis; visualization; project administration; methodology; writing—review and editing. **Hai‐Xi Sun**: Methodology; project administration; supervision. **Xin Wang**: Data curation; formal analysis. **Hang Zhang**: Investigation; validation. **Qiuting Deng**: Investigation. **Yue Yuan**: Investigation. **Chang Liu**: Investigation. **Zhen Wang**: Investigation. **Jiping Li**: Investigation. **Liuwei Xie**: Investigation. **Yujiao Tang**: Investigation. **Jimei Wang**: Investigation. **Chao Ma**: Investigation. **Nan Li**: Investigation. **Pengfei Hu**: Investigation. **Qianqian Guo**: Investigation. **Guokun Zhang**: Investigation. **Dawn Elizabeth Coates**: Writing—review and editing. **Ying Gu**: Supervision; funding acquisition. **Chuanyu Liu**: Supervision; methodology; writing—original draft. **Datao Wang**: Funding acquisition; validation; resources. **Chunyi Li**: Funding acquisition; writing—review and editing; writing—original draft; project administration; supervision; resources; conceptualization. All authors have read the final manuscript and approved it for publication.

## CONFLICT OF INTEREST STATEMENT

The authors declare no conflicts of interest.

## ETHICS STATEMENT

All experimental procedures involving deer and mice were approved by the Ethics Committee of Changchun Sci‐Tech University (Permit Number: CKARI202112).

## Supporting information


**FIGURE S1.** Quality control of snRNA‐seq data.
**FIGURE S2.** snRNA‐seq analysis of five tissue layers in the AGC.
**FIGURE S3.** Quality control of snATAC‐seq data.
**FIGURE S4.** snATAC‐seq analysis of five tissue layers and integrated multi‐omic analysis.
**FIGURE S5.** Quality control of Stereo‐seq data.
**FIGURE S6.** Stereo‐seq analysis of the AGC.
**FIGURE S7.** AnPC‐associated analysis.
**FIGURE S8.** Cross‐tissue snRNA‐seq comparison.
**FIGURE S9.** Spatial analysis of apoptosis‐related genes.
**FIGURE S10.** Pearson correlation between pseudotime values inferred by StaVia and Monocle3.
**FIGURE S11.** Analysis of vascular cells in snRNA‐seq and snATAC‐seq.
**FIGURE S12.** Characterization of *PHEX* ^+^ cells and hedgehog signaling in chondrocytes.

## Data Availability

The data that support the findings of this study are openly available in CNGBdb at https://db.cngb.org/data_resources/?query=CNP0003724, reference number CNP0003724. All data supporting the findings of this study are included in the main text and the supplementary materials. The data that support the findings of this study are openly available in the China National GeneBank Database (CNGBdb): https://db.cngb.org/data_resources/?query=CNP0003724. The data used for the main figures and scripts used in this study can be found on GitHub: https://github.com/heshidian/DeerAntlersSingleCell. Supplementary materials (methods, figures, graphical abstract, slides, videos, Chinese translated version, and update materials) may be found in the online DOI or iMeta Science http://www.imeta.science/.
