# Supplementary material for: A vast stem‐progenitor cell pool, richly vascular system, and hybrid ossification drive the daily centimeter‐scale elongation of bony antlers
Source: Imeta. 2025 Dec 8;4(6):e70097. doi: 10.1002/imt2.70097 (PMC12747533; doi:10.1002/imt2.70097)
Supplement: Supplementary file 1 — FIGURE S1. Quality control of snRNA‐seq data. FIGURE S2. snRNA‐seq analysis of five tissue layers in the AGC. FIGURE S3. Quality control of snATAC‐seq data. FIGURE S4. snATAC‐seq analysis of five tissue layers and integrated multi‐omic analysis. FIGURE S5. Quality control of Stereo‐seq data. FIGURE S6. Stereo‐seq analysis of the AGC. FIGURE S7. AnPC‐associated analysis. FIGURE S8. Cross‐tissue snRNA‐seq comparison. FIGURE S9. Spatial analysis of apoptosis‐related genes. FIGURE S10. Pearson correlation between pseudotime values inferred by StaVia and Monocle3. FIGURE S11. Analysis of vascular cells in snRNA‐seq and snATAC‐seq. FIGURE S12. Characterization of PHEX + cells and hedgehog signaling in chondrocytes. [file IMT2-4-e70097-s001.docx]

**Supporting information to**

**A vast stem-progenitor cell pool, richly vascular system and hybrid ossification drive the daily centimeter-scale elongation of bony antlers**

**Running title**: A unique system drives the centimeter-scale daily elongation of bony antlers

Hengxing Ba^1,2#*^, Shidian He^3,4#^, Hai-Xi Sun^3,4,5,#^, Xin Wang^6,7#^, Hang Zhang^8#^, Qiuting Deng^5^, Yue Yuan^9^, Chang Liu^5,10^, Zhen Wang^1^, Jiping Li^1^, Liuwei Xie^11^, Yujiao Tang^1^, Jimei Wang^6^, Chao Ma^1^, Nan Li^12^, Pengfei Hu^1^, Qianqian Guo^1^, Guokun Zhang^1^, Dawn Elizabeth Coates^13^, Ying Gu^5,7*^, Chuanyu Liu^5,10*^, Datao Wang^8*^ and Chunyi Li^1,2*^

1 Jilin Provincial Key Laboratory of Deer Antler Biology, Institute of Antler Science and Product Technology, Changchun Sci-Tech University, Changchun 130600, China

2 College of Life Sciences, Jilin Agricultural University, Changchun, 130118, China

3 BGI Research, Beijing 102601, China

4 College of Life Sciences, University of Chinese Academy of Sciences, Beijing 100049, China

5 State Key Laboratory of Genome and Multi-omics Technologies, BGI Research, Shenzhen 518083, China

6 BGI-Shenzhen, Shenzhen 518083, China

7 BGI Cell, Shenzhen 518083, China

8 Institute of Special Economic Animals and Plants, Chinese Academy of Agricultural Sciences, Changchun 130112, China

9 State Key Laboratory of Genome and Multi-omics Technologies, BGI Research, Hangzhou 310030, China

10 Shenzhen Proof-of-Concept Center of Digital Cytopathology, BGI Research, Shenzhen 518083, China

11 Department of Police Dog Technology, Criminal Investigation Police University of China, Shenyang 110048, China

12 Department of Stomatology, Shenzhen People's Hospital (Second Clinical Medical School of Jinan University; First Affiliated Hospital of Southern University of Science and Technology), Shenzhen 518020, China

13 Sir John Walsh Research Institute, Faculty of Dentistry, University of Otago, Dunedin, New Zealand

# These authors contributed equally: Hengxing Ba, Shidian He, Hai-Xi Sun, Xin Wang and Hang Zhang

* Correspondence: [lichunyi1959@163.com](mailto:lichunyi1959@163.com) (Chunyi Li), [wangdatao@caas.cn](mailto:(wangdatao@caas.cn)) (Datao Wang), [liuchuanyu@genomics.cn](mailto:(liuchuanyu@genomics.cn)) (Chuanyu Liu), [guying@genomics.cn](mailto:guying@genomics.cn) (Ying Gu),[bahengxing@cstu.edu.cn](mailto:(bahengxing@cstu.edu.cn) (Hengxing Ba).

**Materials and Methods**

**Samples collection**

For sampling AGC tissues used in snRNA-seq and snATAC-seq, tissues were collected from three healthy 2-year-old sika deer approximately 30 days of growth after casting of their previous hard antlers. The distal 8 cm portion of the AGC was excised and sectioned sagittally along the longitudinal axis. Five distinguishable tissue layers of the AGC were immediately identified, dissected and processed as previously described [1]. For Stereo-seq analysis, a relevant area of 1 cm × 2 cm was selected, which is the maximum size that can be accommodated by the technique. To fit this size, a first growing antler (approximately 10 days old) from a healthy 1-year-old sika deer was used, allowing the 1 cm × 2 cm section to encompass all five tissue layers along with the underlying bone tissue. Following layer dissection, AGC tissues were embedded in Tissue-Tek OCT Compound (Sakura Finetek), respectively, snap-frozen in liquid-nitrogen–precooled isopentane, and stored at −80 °C prior to Stereo-seq. Cryosections were prepared at a thickness of 10 μm using a cryostat (Leica CM3050 S, Leica Biosystems).

**Cell dissociation from AGC tissues**

Each tissue sample was minced into pieces smaller than 1 mm³ and transferred to a 50-mL tube. Samples were digested in DMEM (Sigma-Aldrich) containing 100 μg/mL collagenase types I and II (Invitrogen) at 37 °C for 60-100 minutes with gentle shaking. Digestion was stopped by adding 10% fetal bovine serum (Gibco) after the release of over 50,000 cells. The cell suspension was filtered through a 70-μm strainer, centrifuged at 500 g for 5 minutes at 4 °C, and treated with 1× RBC lysis buffer (Beyotime) to remove erythrocytes. Cells were washed with PBS, and viable cells were counted using an AO/PI staining kit (Beyotime).

**snRNA-seq and snATAC-seq library preparation and sequencing**

snRNA-seq libraries were prepared using the DNBelab C Series Single-Cell Library Prep Set (MGI, catalog #1000021082) according to established protocols. Briefly, single-cell suspensions were partitioned into individual droplets using droplet-based microfluidics. After emulsion breakage, mRNA-captured beads were collected, followed by reverse transcription, cDNA amplification, and purification.

snATAC-seq libraries were constructed using the DNBelab C Series snATAC Library Prep Set (MGI, catalog #1000021878). One library was generated per 10,000 viable nuclei, following the manufacturer’s protocol. Transposed single-nucleus suspensions were partitioned via droplet-based microfluidics, preamplified within droplets, and then the emulsions were disrupted. Barcoded DNA was subsequently amplified and purified to generate sequencing libraries.

Library quality and concentration for both snRNA-seq and snATAC-seq were assessed using the Qubit ssDNA Assay Kit (Thermo Fisher Scientific, Q10212). Sequencing was performed on different platforms according to library type. snRNA-seq libraries were sequenced on the DIPSEQ T1 platform at China National GeneBank (CNGB) using paired-end 130 bp reads: Read 1 (30 bp) included two 10 bp cell barcodes and a 10 bp unique molecular identifier (UMI), and Read 2 (100 bp) captured the transcript sequence, with an additional 10 bp sample index. snATAC-seq libraries were sequenced on the BGISEQ-500 platform with 50 bp paired-end reads. Stringent quality control procedures were maintained throughout the library preparation and sequencing workflow to ensure data integrity.

**snRNA-seq data pre-processing**

First, PISA (v.1.1) [2] was used to extract bead barcodes and unique molecular identifier (UMI) sequences from the raw sequencing data. Processed reads were then aligned to the reference genome [3] using STAR (v.2.5.3) [4]. To ensure data quality, beads with UMI counts below the defined threshold were filtered out, and barcodes representing the same cell were merged. Finally, gene expression profiles were quantified for each barcode using PISA.

**snATAC-seq data pre-processing**

Raw sequencing reads were first processed using PISA to extract bead barcodes and unique molecular identifiers (UMIs). Reads were then aligned to the reference genome [3] using BWA (v.0.7.15) [5] to generate BAM files. The resulting BAM files were further processed with bap2 (v.0.6.2) [6] to produce fragment files for each snATAC-seq library.

**Computational analysis of snRNA-seq data**

Gene-barcode expression count matrices were loaded into R (v.4.4.1) and converted to Seurat objects for downstream analysis using the Seurat package (v.4.4.0) [7]. Poor-quality cells, defined as those detecting fewer than 200 genes, and genes detected in fewer than 3 cells, were filtered out. Potential doublets were identified and excluded using DoubletFinder (v.2.0.3) [8] to minimize the impact of technical artifacts on the analysis. Cells with a high mitochondrial gene percentage (> 5%) were also excluded, as these may indicate apoptosis or cell lysis. After quality control, a total of 56,254 high-quality barcodes were retained for downstream analysis. Log-normalization was conducted using the ‘NormalizeData’ function, which scaled each cell’s total read count to 10,000. The top 2,000 highly variable genes were identified using the ‘FindVariableFeatures’ function with the ‘vst’ method. Gene expression for each cell was then standardized using the ‘ScaleData’ function. Dimensionality reduction was performed using principal component analysis (PCA). Batch effects across samples were corrected using the Harmony (v.1.2.0) [9] algorithm. The Harmony-generated embeddings were used for clustering by constructing a shared nearest-neighbor graph. The clusters identified by the Louvain algorithm were evaluated for the expression patterns of well-known cell-type marker genes. The ‘CellCycleScoring’ function from Seurat was used to assign a cell-cycle score to each cell and predict its phase (G2M, S, or G1).

**Computational analysis of snATAC-seq data**

Downstream processing of snATAC-seq data was performed using the ArchR package (v.1.0.2) [10]. For each sample, an Arrow file was generated and subsequently integrated into an ArchRProject object. Genome and gene annotations were constructed using the ‘createGenomeAnnotation’ and ‘createGeneAnnotation’ functions, respectively. Mitochondrial fragments were excluded, and quality metrics were computed for each nucleus. Nuclei with a transcription start site (TSS) enrichment score < 1, unique fragment count < 1,000, or nucleosome signal > 2 were removed. Potential doublets were identified using ‘addDoubletScores’ (nTrials = 20, k = 10, knnMethod = 'UMAP', dimsToUse = 1:30) and filtered with ‘filterDoublets’ (filterRatio = 1). After quality control, 103,278 nuclei remained. Gene-level chromatin accessibility was assessed using the ‘addGeneScoreMatrix’ function. Dimensionality reduction was conducted via iterative latent semantic indexing (LSI) on the TileMatrix with ‘addIterativeLSI’. The top 30 LSI components were corrected for batch effects using Harmony. Cell clustering was performed with ‘addClusters’ (resolution = 1.2, dimsToUse = 1:30, maxClusters = 50), and cluster-specific activated genes were identified using ‘getMarkerFeatures’ (useMatrix = 'GeneScoreMatrix', bias = c('TSSEnrichment','log10(nFrags)'), testMethod = 'wilcoxon'). Cell type annotation was based on markers used for snRNA-seq, and UMAP visualization was generated via addUMAP. For high-precision integration with snRNA-seq data, the ‘addGeneIntegrationMatrix’ function was applied. Cell types were grouped into four categories: (1) AnSCs, AnPCs, proliferative AnPCs, and mural cells; (2) chondrocytes; (3) endothelial cells, monocytes/macrophages, mast cells, and chondroclasts; and (4) hypertrophic chondrocytes. Within each group, cells were stratified by tissue layers (RM, PC, TZ, CA, and MC) to account for biological heterogeneity. Each snATAC-seq cell was assigned a predicted RNA cell barcode, generating an integrated gene expression profile stored in ‘GeneIntegrationMatrix’. Pseudo-bulk replicates at the cell type level were generated using ‘addGroupCoverages’, and reproducible, fixed-width peaks (501 bp) were called with ‘addReproduciblePeakSet’ using MACS2 [11]. Peaks were annotated relative to the nearest gene and classified as promoter, distal, exonic, or intronic. Human TF binding motifs were obtained from CIS-BP (v.2.0.0) [12], annotated using ‘addMotifAnnotations’, and motif activity in individual cells was calculated with ‘addDeviationsMatrix’, stored in ‘MotifMatrix’.

**Stereo-seq chip preparation**

Tissue sections were mounted onto the Stereo-seq chip (BGI) and incubated at 37 °C for 3 minutes. Sections were subsequently fixed in methanol at −20 °C for 30 minutes. After fixation, the sections were washed with 0.1× SSC buffer (Thermo) containing RNase inhibitor (NEB), followed by permeabilization with 0.1% pepsin (Sigma) in 0.01 M HCl at 37 °C for 5 minutes. RNA molecules were captured by DNA nanoballs (DNBs) and reverse transcribed overnight at 42 °C using SuperScript II (Invitrogen) with appropriate reagents. After reverse transcription, tissue sections were washed and digested with Tissue Removal buffer at 55 °C for 10 minutes. The released cDNA was purified using VAHTS™ DNA Clean Beads (0.8×) and subsequently amplified using KAPA HiFi HotStart ReadyMix (Roche) with the cDNA-PCR primer. PCR conditions were: 95 °C for 5 minutes, 15 cycles of 98 °C for 20 seconds, 58 °C for 20 seconds, 72 °C for 3 minutes, and a final extension at 72 °C for 5 minutes.

**Stereo-seq library preparation and sequencing**

The concentration of amplified cDNA was measured using the Qubit™ dsDNA Assay Kit (Thermo Fisher). A total of 20 ng of DNA was fragmented using in-house Tn5 transposase at 55 °C for 10 minutes. The reaction was terminated by adding 0.02% SDS, followed by gentle mixing at 37 °C for 5 minutes. For library amplification, 25 μL of fragmented DNA was combined with 1× KAPA HiFi HotStart ReadyMix, 0.3 mM Stereo-seq-Library-F primer, 0.3 mM Stereo-seq-Library-R primer, and nuclease-free water to a final volume of 100 μL. PCR was performed under the following conditions: 95 °C for 5 minutes; 13 cycles of 98 °C for 20 seconds, 58 °C for 20 seconds, and 72 °C for 30 seconds; followed by a final extension at 72 °C for 5 minutes. The PCR products were purified using AMPure XP beads at bead-to-sample ratios of 0.63× and 0.153×. Purified libraries were then used for DNA nanoball (DNB) generation and sequenced on the MGI DNBSEQ-Tx platform (Novogene).

**Stereo-seq data processing and annotation**

FASTQ files generated by the sequencer were processed using the publicly available SAW software suite (v.7.1; [https://github.com/STOmics/SAW](https://github.com/STOmics/SAW?utm_source=chatgpt.com)). Following the standard SAW workflow, spatial gene expression matrices were generated in GEF format. The raw spatial expression matrix was aggregated into 100 × 100 DNB bins, each representing approximately one cell, resulting in a median detection of 5,430 mRNA molecules and 1,166 genes per spot. Downstream analysis was performed using the Seurat framework. Molecular counts were normalized and variance-stabilized using SCTransform v2, generating the “SCT” assay. Principal component analysis (PCA) was applied, and the first 30 principal components were used for cluster identification with the Louvain algorithm at a resolution of 0.935.

**Cell-Cell communication inference**

Cell-cell communication networks were inferred using the CellChat (v2.1.2) toolkit [13] based on both single-cell transcriptomic and spatial transcriptomic datasets. Communication probabilities between cell populations were calculated with the ‘computeCommunProb’ function, and significant ligand–receptor (LR) interactions were visualized using the ‘netVisual_bubble’ function. To assess dynamic changes in signaling, communication probabilities were compared between relevant cellular subgroups, and differential probabilities (delta values) were computed and displayed as dot plots.

**Identification of activated TFs and their target genes in proliferative and non-proliferative AnPCs**

To identify TFs with potential regulatory roles in proliferative versus non-proliferative AnPCs, we first performed differential analysis of TF motif activity and TF expression using the ‘getMarkerFeatures’ function. Specifically, TF motif activity was assessed by setting useMatrix = ‘MotifMatrix’, while TF expression was evaluated by setting useMatrix = ‘GeneIntegrationMatrix’. TFs were considered enriched in proliferative AnPCs if they exhibited a log_2_ fold change (log_2_FC) in expression ≥ 1 and an area under the curve (AUC) score ≥ 0.6. Conversely, TFs enriched in non-proliferative AnPCs were identified if their log_2_FC ≤ −1 and AUC ≤ 0.4.

Next, we sought to identify downstream target genes of the enriched TFs by integrating chromatin accessibility and gene expression data. Gene expression imputation was performed using the ‘imputeMatrix’ function with the Markov Affinity-based Graph Imputation of Cells (MAGIC) algorithm. Differentially accessible peaks between proliferative and non-proliferative AnPCs were identified using the ‘getMarkerFeatures’ function (with useMatrix = "PeakMatrix"), applying thresholds of log_2_FC ≥ 1 and *p* < 0.05 for proliferative AnPCs, and log_2_FC ≤ −1 and *p* < 0.05 for non-proliferative AnPCs. A TF was considered functionally linked to a downstream gene if the corresponding motif was able to bind to peaks (located in the promoter or distal regulatory regions) that were differentially accessible in the relevant AnPC population, and if the TF-target gene pair exhibited a significant positive correlation in gene expression (Pearson’s r > 0.3, *p* < 0.05).

We also used the SCENIC pipeline [14] to infer TF-target regulatory relationships in proliferative and non-proliferative AnPCs, obtaining regulons associated with the top five activated TFs in each group. For each regulon, we calculated its activity across cells using AUCell (v.1.24.0).

**Enrichment analysis**

To gain insights into the biological functions associated with the activated TFs in proliferative AnPCs and their potential targets, we performed functional enrichment analysis (GO and KEGG) using the DAVID (v.2023q4) database [15]. We also used Cytoscape (v.3.9.0) [16] to construct a network visualizing TF-target regulatory relationships and enriched annotations.

**Processing of published datasets**

The scRNA-seq expression profiles of human adult intervertebral discs (ID) were obtained from the Gene Expression Omnibus (GEO; accession number GSE160756) [17]. Low-quality cells were removed if they had fewer than 300 or more than 4,000 detected genes, fewer than 2,000 or more than 20,000 UMIs, or were apoptotic/lysed (mitochondrial gene percentage > 5% or ribosomal gene percentage > 40%). Data normalization and identification of highly variable features were performed, followed by dimensionality reduction using PCA. Batch effects were corrected with Harmony based on the first 50 principal components. UMAP was used for visualization, and clustering was performed at a resolution of 0.3. Cell type annotation was based on marker genes reported in the original publication.

The scRNA-seq datasets of human embryonic long bones (EL) were downloaded from GEO (GSE143753) [18]. Low-quality cells were excluded if they had fewer than 300 or more than 6,000 detected genes, fewer than 2,000 or more than 40,000 UMIs, or were apoptotic/lysed (mitochondrial > 5% or ribosomal > 60%). Normalization, identification of highly variable features, PCA-based dimensionality reduction, and batch correction with Harmony (first 50 PCs) were applied. UMAP visualization and clustering analysis were conducted at a resolution of 0.13, with cell types annotated using published marker genes.

The scRNA-seq datasets of osteosarcoma (Os) were obtained from GEO (GSE152048) [19]. Poor-quality nuclei were filtered out if they had fewer than 300 or more than 8,000 detected genes, fewer than 2,000 or more than 50,000 UMIs, or were apoptotic/lysing (mitochondrial > 10% or ribosomal > 40%). Normalization, identification of highly variable features, PCA, and Harmony-based batch correction (first 50 PCs) were applied. UMAP visualization and clustering (resolution = 0.15) were performed, and cell type annotation was based on the original publication’s markers.

**Comparison of transcriptome similar probability of AnSC-derived cells with published datasets**

We employed ScPred v1.9.2 [20], which uses all principal components as the gene feature space, to train classifiers with AnSC-derived cells as reference. By default, the models were implemented as support vector machines with a radial kernel. Using these classifiers, we subsequently predicted the transcriptome similarity probabilities of cell types in ID, EL, and Os by comparing them to the reference cell types.

**Gene set activity scoring**

To assess the activity of GSEA hallmark gene sets in the snRNA-seq dataset, we employed the AUCell (v.1.24.0) package to calculate the AUC score for each cell. For the snATAC-seq dataset, gene set activity scores were quantified with the addModuleScore function in ArchR, which evaluates the accessibility of genomic regions associated with predefined gene sets and summarizes them into per-cell activity scores.

**Identification of activated MYC target genes in AGC AnPCs and Os cell line**

The scATAC-seq dataset of Os cell line (MG63.3-GFP cells) was obtained from GEO (GSE215758) [21]. Authentication, source information, and mycoplasma testing were conducted by the original authors. As our study relied exclusively on this publicly available dataset, no additional cell line validation was required. Low-quality cells with a TSS enrichment score < 1 or fewer than 1,000 total fragments were excluded. Dimensionality reduction, clustering, and peak calling were subsequently performed using the ArchR package. In both AnPCs and Os cell line, the activated *MYC* target genes were identified based on two complementary criteria: (1) the regulatory region (promoter or distal peaks) of a candidate gene contains *MYC* binding motifs, and the chromatin accessibility of this region shows a significant positive correlation with the gene’s expression (Pearson correlation’s r > 0.3, *p* < 0.05); and (2) *MYC* expression itself is significantly positively correlated with the expression of the candidate gene (Pearson correlation’s r > 0.3, *p* < 0.05). Genes fulfilling both criteria were defined as high-confidence activated MYC target genes.

**Trajectory analysis**

Cellular trajectories were inferred independently using Monocle3 (v1.3.4) [90] and StaVia (Via 2.0) [22]. Monocle3 reconstructed lineage progression by applying principal graph learning on the UMAP embedding, whereas StaVia employed higher-order lazy-teleporting random walks with memory to capture both local transitions and global topology. Fine-grained vector fields generated by StaVia were visualized with the ‘via_streamplot’ function to illustrate the flow of cellular state transitions. Concordance between the two approaches was assessed by calculating the Pearson correlation of pseudotime values across all shared cells.

**Definition of activated and quiescent states of vascular cells**

We applied the CytoTRACE2 (v.1.0.0) [23] algorithm to evaluate the differentiation potential of endothelial and mural cells based on their gene expression profiles, where higher scores indicate a more progenitor-like and transcriptionally active state. Cells were subsequently stratified into activated and quiescent states using a fixed threshold of 0.3, derived from the overall score distribution across regions. Cells with scores above this threshold were classified as activated, whereas those with lower scores were designated as quiescent.

**Identification of activated TFs and their target genes in activated and quiescent states of both endothelial and mural cells**

To identify activated TFs in endothelial and mural cells under activated and quiescent states, we integrated the snATAC-seq and snRNA-seq datasets using the ArchR function addGeneIntegrationMatrix. Each snATAC-seq cell was assigned the label of its most transcriptionally similar neighbor from the snRNA-seq dataset, enabling classification of endothelial and mural cells in the snATAC-seq dataset into activated and quiescent states.

Activated TFs were identified by integrating transcriptional and chromatin accessibility data. Differential gene expression between activated and quiescent states was assessed using the FindMarkers function in Seurat, while differential chromatin accessibility was computed with the getMarkerFeatures function in ArchR (useMatrix = "GeneScoreMatrix"). TFs with positive log_2_FC in both transcriptional expression and chromatin accessibility were classified as activated in the activated state, whereas those with negative log_2_FC in both modalities were designated as activated in the quiescent state. Downstream target genes of activated TFs were inferred as described in the section “Identification of activated TF and their target genes in proliferative and non-proliferative AnPCs.”

**Co-culture of AnSCs and AnPCs**

The *THY1*^+^*RXFP2*^+^ AnSCs [24] and *TNN*^+^*TNC*^+^ AnPCs [25] were isolated from AGC as previously described. All cells were cultured in Dulbecco's Modified Eagle Medium (DMEM; Gibco) supplemented with 100 U/mL penicillin, 100 μg/mL streptomycin, and 10% fetal bovine serum (FBS; HyClone), in a humidified incubator at 37 °C with 5% CO_2_. For the co-culture assay, AnPCs were seeded in the lower chamber, while AnSCs were seeded in the upper chamber of transwell inserts with 0.4 μm pores. The co-culture was maintained for one week under standard culture conditions (37 °C, 5% CO_2_).

**Assessment of AnPC viability and proliferation**

Following co-culture, AnPCs were subjected to cell viability analysis using the Cell Counting Kit-8 (CCK-8, Cat. No. K1018, Apexbio). Viability was determined by measuring absorbance and comparing it to that of untreated control cells. In addition, cell proliferation was quantified by immunofluorescence staining for Ki67, a nuclear marker of proliferating cells. Briefly, AnPCs were fixed with 4% paraformaldehyde, permeabilized with 0.1% Triton X-100, and incubated with an Ki67 primary antibody (1:200, AB15580, abcam) followed by an appropriate fluorescent secondary antibody. Nuclei were counterstained with DAPI. The proportion of Ki67-positive cells relative to total nuclei was calculated to quantify proliferation.

**Immunohistochemistry**

Paraffin-embedded AGC tissue sections (2-3 mm in thickness) were de-paraffinized and rehydrated. Endogenous peroxidase was quenched with 3% H_2_O_2_ for 5 min. Antigen retrieval was performed by boiling in 10 mM sodium citrate buffer (pH 6.0) for 10 min. The non-specifc binding sites were blocked in PBS plus 10% normal goat serum for 30 min and then incubated with primary antibodies: RUNX2, (1:500, cat. No. 20700-1-AP, Proteintech), MCAM (1:200, A13927, ABclonal) and CASP3 (1:200, abs119676, absin) at 37 °C for 2 hour. After rinsing with PBS, sections were incubated with secondary antibody for 30 min. After rinsing in PBS, all sections were stained with DAB chromogen reaction solution. TUNEL (C1089, Beyotime) assay according to the manufacturer’s instructions.

**Immunofluorescence staining**

Frozen tissue samples were cryosectioned at a thickness of 10 μm, thawed, and washed with PBS. To block non-specific binding, sections were incubated for 1 hour in PBS containing 10% goat serum and 0.3% Triton X-100. Primary antibodies (CD31, 1:1000, ab281583, Abcam; α-Smooth Muscle Actin, 1:500, ab7817, Abcam; BGLAP, 1:200, A20800, ABclonal; SP7, 1:500, ab209484, Abcam) were diluted in PBS and applied to the sections overnight at 4 °C. After washing three times with PBS, sections were incubated with secondary antibodies for 1 hour at room temperature. Sections were then washed with PBS for 3 minutes and counterstained with DAPI (1:500) for 5 minutes. Fluorescence images were acquired using an EVOS M5000 microscope (Thermo Fisher).

**Creation of ectopic antlers either with vascularized or with avascularized cartilage on the nude mice foreheads**

A 9-month-old male sika deer calf was selected prior to pedicle initiation (March) for antler stem cell tissue (antlerogenic periosteum, AP) collection and transplantation into nude mice, aiming to generate two types of ectopic antlers: one comprising vascularized cartilage (VC-antler) in red fluorescent protein (RFP)-expressing nude mice, and the other comprising avascular cartilage (AC-antler) in conventional nude mice. The AP from one antler was divided into six pieces for VC-antler induction, and the AP from the contralateral side was similarly divided for AC-antler formation. Detailed surgical procedures were described in our previous studies [26-28]. Briefly, to induce VC-antlers, the periosteum at the implantation site on each mouse skull was completely removed prior to subcutaneous implantation of the AP. In contrast, to induce AC-antlers, the periosteum was left intact. In the absence of host periosteum, the implanted AP fused more effectively with the underlying periosteum-free skull, leading to the formation of larger ectopic antlers with vascularized cartilage. When the host periosteum was preserved, fusion was inhibited, resulting in smaller ectopic antlers with avascular cartilage.

**Statistical and Reproducibility**

The statistical methods used are indicated in the figure legends. All calculations and visualizations were performed in R. No statistical methods were used to predetermine the sample size, and the experiment was not randomized.

**References**

1. Li, Chunyi, Dawn E. Clark, Eric A. Lord, Jo-Anne L. Stanton, James M. Suttie. 2002. “Sampling technique to discriminate the different tissue layers of growing antler tips for gene discovery.” *The Anatomical Record* 268: 125-130. <https://doi.org/https://doi.org/10.1002/ar.10120>

2. Shi, Quan, Shiping Liu, Karsten Kristiansen, Longqi Liu. 2022. “The FASTQ+ format and PISA.” *Bioinformatics* 38: 4639-4642. <https://doi.org/10.1093/bioinformatics/btac562>

3. Ba, Hengxing, Zexi Cai, Haoyang Gao, Tao Qin, Wenyuan Liu, Liuwei Xie, Yaolei Zhang, et al. 2020. “Chromosome-level genome assembly of Tarim red deer, Cervus elaphus yarkandensis.” *Scientific Data* 7: 187. <https://doi.org/10.1038/s41597-020-0537-0>

4. Dobin, Alexander, Carrie A. Davis, Felix Schlesinger, Jorg Drenkow, Chris Zaleski, Sonali Jha, Philippe Batut, et al. 2012. “STAR: ultrafast universal RNA-seq aligner.” *Bioinformatics* 29: 15-21. <https://doi.org/10.1093/bioinformatics/bts635>

5. Li, Heng. 2014. “Toward better understanding of artifacts in variant calling from high-coverage samples.” *Bioinformatics* 30: 2843-2851. <https://doi.org/10.1093/bioinformatics/btu356>

6. Lareau, Caleb A., Fabiana M. Duarte, Jennifer G. Chew, Vinay K. Kartha, Zach D. Burkett, Andrew S. Kohlway, Dmitry Pokholok, et al. 2019. “Droplet-based combinatorial indexing for massive-scale single-cell chromatin accessibility.” *Nature Biotechnology* 37: 916-924. <https://doi.org/10.1038/s41587-019-0147-6>

7. Hao, Yuhan, Stephanie Hao, Erica Andersen-Nissen, William M. Mauck, III, Shiwei Zheng, Andrew Butler, Maddie J. Lee, et al. 2021. “Integrated analysis of multimodal single-cell data.” *Cell* 184: 3573-3587.e3529. <https://doi.org/10.1016/j.cell.2021.04.048>

8. McGinnis, Christopher S., Lyndsay M. Murrow, Zev J. Gartner. 2019. “DoubletFinder: Doublet Detection in Single-Cell RNA Sequencing Data Using Artificial Nearest Neighbors.” *Cell Systems* 8: 329-337.e324. <https://doi.org/10.1016/j.cels.2019.03.003>

9. Korsunsky, Ilya, Nghia Millard, Jean Fan, Kamil Slowikowski, Fan Zhang, Kevin Wei, Yuriy Baglaenko, et al. 2019. “Fast, sensitive and accurate integration of single-cell data with Harmony.” *Nature Methods* 16: 1289-1296. <https://doi.org/10.1038/s41592-019-0619-0>

10. Granja, Jeffrey M., M. Ryan Corces, Sarah E. Pierce, S. Tansu Bagdatli, Hani Choudhry, Howard Y. Chang, William J. Greenleaf. 2021. “ArchR is a scalable software package for integrative single-cell chromatin accessibility analysis.” *Nature Genetics* 53: 403-411. <https://doi.org/10.1038/s41588-021-00790-6>

11. Zhang, Yong, Tao Liu, Clifford A. Meyer, Jérôme Eeckhoute, David S. Johnson, Bradley E. Bernstein, Chad Nusbaum, et al. 2008. “Model-based Analysis of ChIP-Seq (MACS).” *Genome Biology* 9: R137. <https://doi.org/10.1186/gb-2008-9-9-r137>

12. Weirauch, Matthew T, Ally Yang, Mihai Albu, Atina G. Cote, Alejandro Montenegro-Montero, Philipp Drewe, Hamed S Najafabadi, et al. 2014. “Determination and inference of eukaryotic transcription factor sequence specificity.” *Cell* 158: 1431-1443. <https://doi.org/10.1016/j.cell.2014.08.009>

13. Jin, Suoqin, Maksim V. Plikus, Qing Nie. 2025. “CellChat for systematic analysis of cell–cell communication from single-cell transcriptomics.” *Nature Protocols* 20: 180-219. <https://doi.org/10.1038/s41596-024-01045-4>

14. Aibar, Sara, Carmen Bravo González-Blas, Thomas Moerman, Vân Anh Huynh-Thu, Hana Imrichova, Gert Hulselmans, Florian Rambow, et al. 2017. “SCENIC: single-cell regulatory network inference and clustering.” *Nature Methods* 14: 1083-1086. <https://doi.org/10.1038/nmeth.4463>

15. Sherman, Brad T, Ganesh Panzade, Tomozumi Imamichi, Weizhong Chang. 2024. “DAVID Ortholog: an integrative tool to enhance functional analysis through orthologs.” *Bioinformatics* 40: <https://doi.org/10.1093/bioinformatics/btae615>

16. Shannon, Paul, Andrew Markiel, Owen Ozier, Nitin S Baliga, Jonathan T Wang, Daniel Ramage, Nada Amin, et al. 2003. “Cytoscape: a software environment for integrated models of biomolecular interaction networks.” *Genome Res* 13: 2498-2504. <https://doi.org/10.1101/gr.1239303>

17. Gan, Yibo, Jian He, Jun Zhu, Zhengyang Xu, Zhong Wang, Jing Yan, Ou Hu, et al. 2021. “Spatially defined single-cell transcriptional profiling characterizes diverse chondrocyte subtypes and nucleus pulposus progenitors in human intervertebral discs.” *Bone Research* 9: 37. <https://doi.org/10.1038/s41413-021-00163-z>

18. He, Jian, Jing Yan, Jianfang Wang, Liangyu Zhao, Qian Xin, Yang Zeng, Yuxi Sun, et al. 2021. “Dissecting human embryonic skeletal stem cell ontogeny by single-cell transcriptomic and functional analyses.” *Cell Research* 31: 742-757. <https://doi.org/10.1038/s41422-021-00467-z>

19. Zhou, Yan, Dong Yang, Qingcheng Yang, Xiaobin Lv, Wentao Huang, Zhenhua Zhou, Yaling Wang, et al. 2020. “Single-cell RNA landscape of intratumoral heterogeneity and immunosuppressive microenvironment in advanced osteosarcoma.” *Nature Communications* 11: 6322. <https://doi.org/10.1038/s41467-020-20059-6>

20. Alquicira-Hernandez, Jose, Anuja Sathe, Hanlee P. Ji, Quan Nguyen, Joseph E. Powell. 2019. “scPred: accurate supervised method for cell-type classification from single-cell RNA-seq data.” *Genome Biology* 20: 264. <https://doi.org/10.1186/s13059-019-1862-5>

21. Pontius, W. Dean, Ellen S. Hong, Zachary J. Faber, Jeremy Gray, Craig D. Peacock, Ian Bayles, Katreya Lovrenert, et al. 2023. “Temporal chromatin accessibility changes define transcriptional states essential for osteosarcoma metastasis.” *Nature Communications* 14: 7209. <https://doi.org/10.1038/s41467-023-42656-x>

22. Stassen, Shobana V., Minato Kobashi, Edmund Y. Lam, Yuanhua Huang, Joshua W. K. Ho, Kevin K. Tsia. 2024. “StaVia: spatially and temporally aware cartography with higher-order random walks for cell atlases.” *Genome Biology* 25: 224. <https://doi.org/10.1186/s13059-024-03347-y>

23. Kang, Minji, Gunsagar S. Gulati, Erin L. Brown, Zhen Qi, Susanna Avagyan, Jose Juan Almagro Armenteros, Rachel Gleyzer, et al. 2025. “Improved reconstruction of single-cell developmental potential with CytoTRACE 2.” *Nature Methods* 22: 2258-2263. <https://doi.org/10.1038/s41592-025-02857-2>

24. Ba, Hengxing, Pengfei Hu, Hongming Yuan, Chao Ma, Zhen Wang, Yudong Shang, Qianqian Guo, et al. 2025. "RXFP2-positive mesenchymal stem cells in the antlerogenic periosteum contribute to postnatal development of deer antlers.” *Communications Biology* 8: 645. <https://doi.org/10.1038/s42003-025-08085-w>

25. Qin, Tao, Guokun Zhang, Yi Zheng, Shengyou Li, Yuan Yuan, Qingjie Li, Mingliang Hu, et al. 2023. “A population of stem cells with strong regenerative potential discovered in deer antlers.” *Science* 379: 840-847. <https://doi.org/doi:10.1126/science.add0488>

26. Li, C., J. M. Suttie. 2003. “Tissue collection methods for antler research.” *European Journal of Morphology* 41: 23-30. <https://doi.org/10.1076/ejom.41.1.23.28106>

27. Li, Chunyi, Xiuhua Gao, Fuhe Yang, Shirley K. Martin, Stephen R. Haines, Xuming Deng, John Schofield, et al. 2009. “Development of a nude mouse model for the study of antlerogenesis—mechanism of tissue interactions and ossification pathway.” *Journal of Experimental Zoology Part B*: *Molecular and Developmental Evolution* 312B: 118-135. <https://doi.org/https://doi.org/10.1002/jez.b.21252>

28. Li, Chunyi, A. John Harris, James M. Suttie. 2001. “Tissue interactions and antlerogenesis: New findings revealed by a xenograft approach.” *Journal of Experimental Zoology* 290: 18-30. <https://doi.org/https://doi.org/10.1002/jez.1032>

**
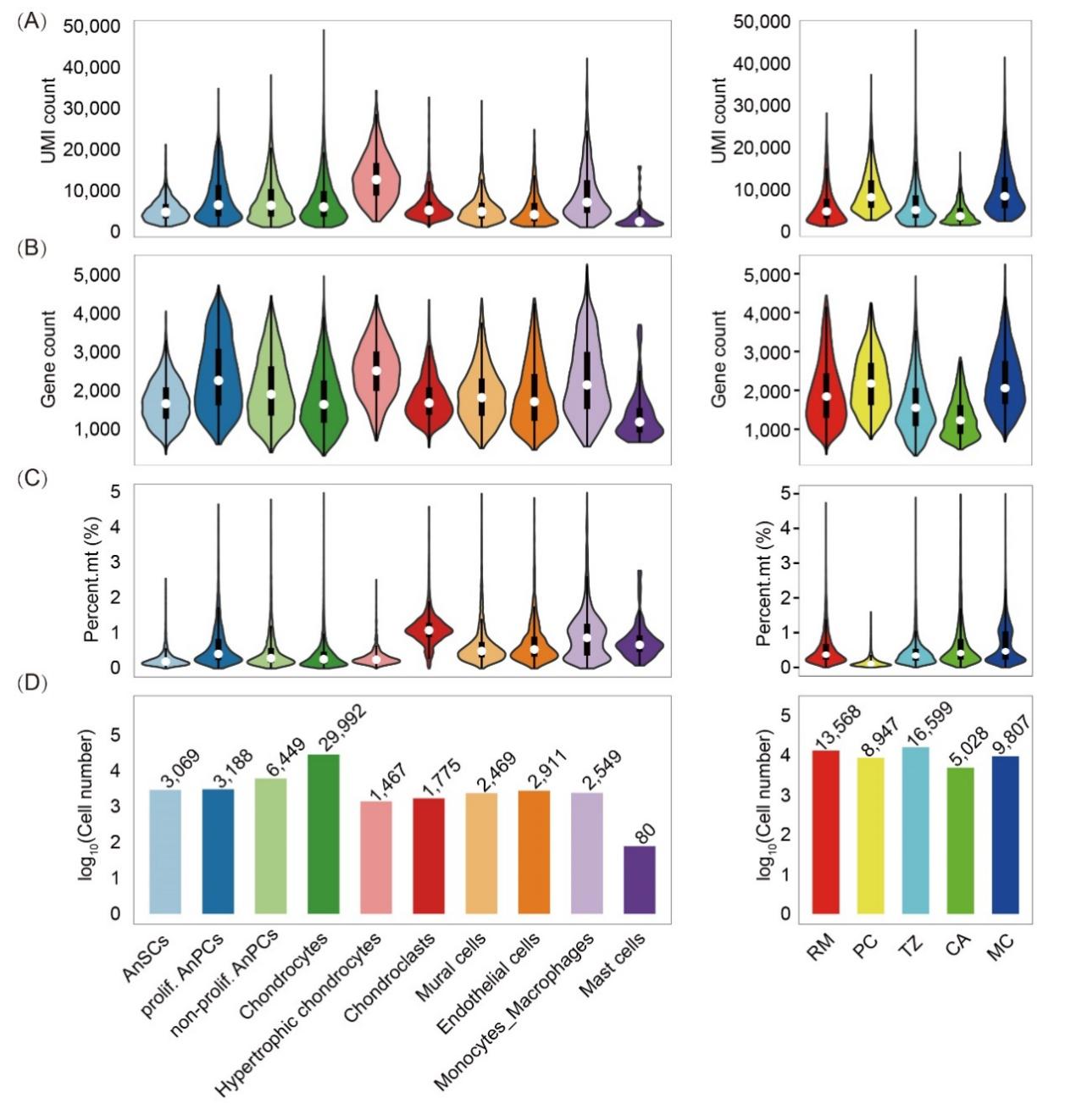
**

**FIGURE S1. Quality control of snRNA-seq data.** (A) Violin plot showing UMI counts for each cell type (left) and tissue layer (right) after stringent quality-control filtering. (B) Violin plot showing the number of detected genes per cell for each cell type (left) and tissue layer (right) after filtering. (C) Violin plot showing the percentage of mitochondrial transcripts (Percent.mt) for each cell type (left) and tissue layer (right) after filtering. (D) Bar plot showing the number of cells retained for each cell type (left) and tissue layer (right) after filtering. AnSCs, antler stem cells; AnPCs, antler progenitor cells; RM, reserve mesenchyme; PC, pre-cartilage; TZ, transition zone; CA, cartilage; MC, mineralized cartilage.

**
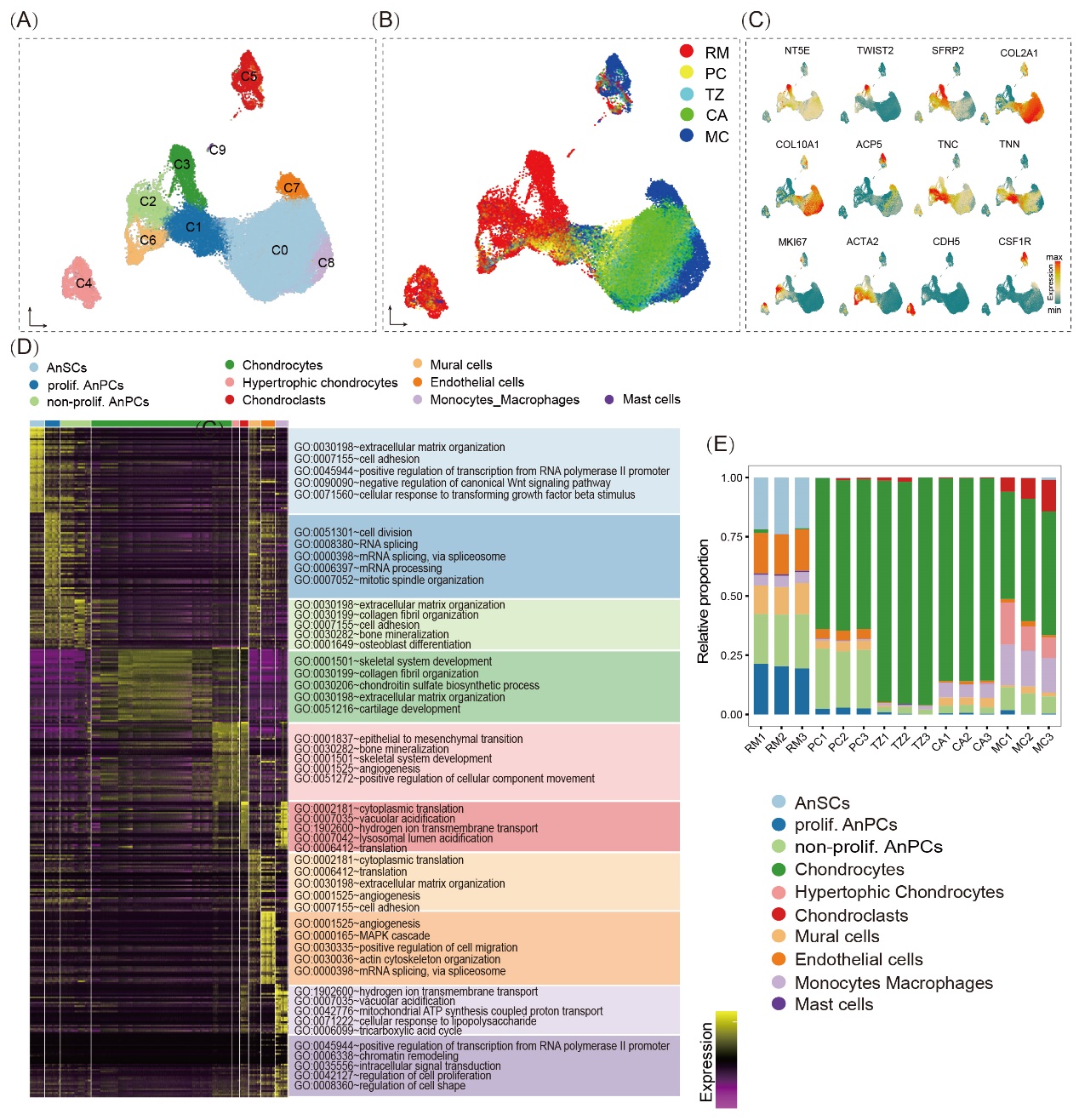
**

**FIGURE S2. snRNA-seq analysis of five tissue layers in the AGC.** (A) UMAP plot of snRNA-seq data showing unsupervised clustering of all cells from the five tissue layers. (B) UMAP plot of snRNA-seq data showing all cells from the five layers, color-coded by tissue layer. (C) UMAP plots showing the expression patterns of representative marker genes for the 10 identified cell types. (D) Heatmap illustrating the expression of the top 50 highly expressed genes in all cell types. DAVID GO-enriched biological processes (adjusted *p* < 0.05) are listed on the right. (E) Bar plot showing inferred proportions of each cell type across the five layers based on deconvolution of bulk RNA-seq data (Ba et al., 2019).

**
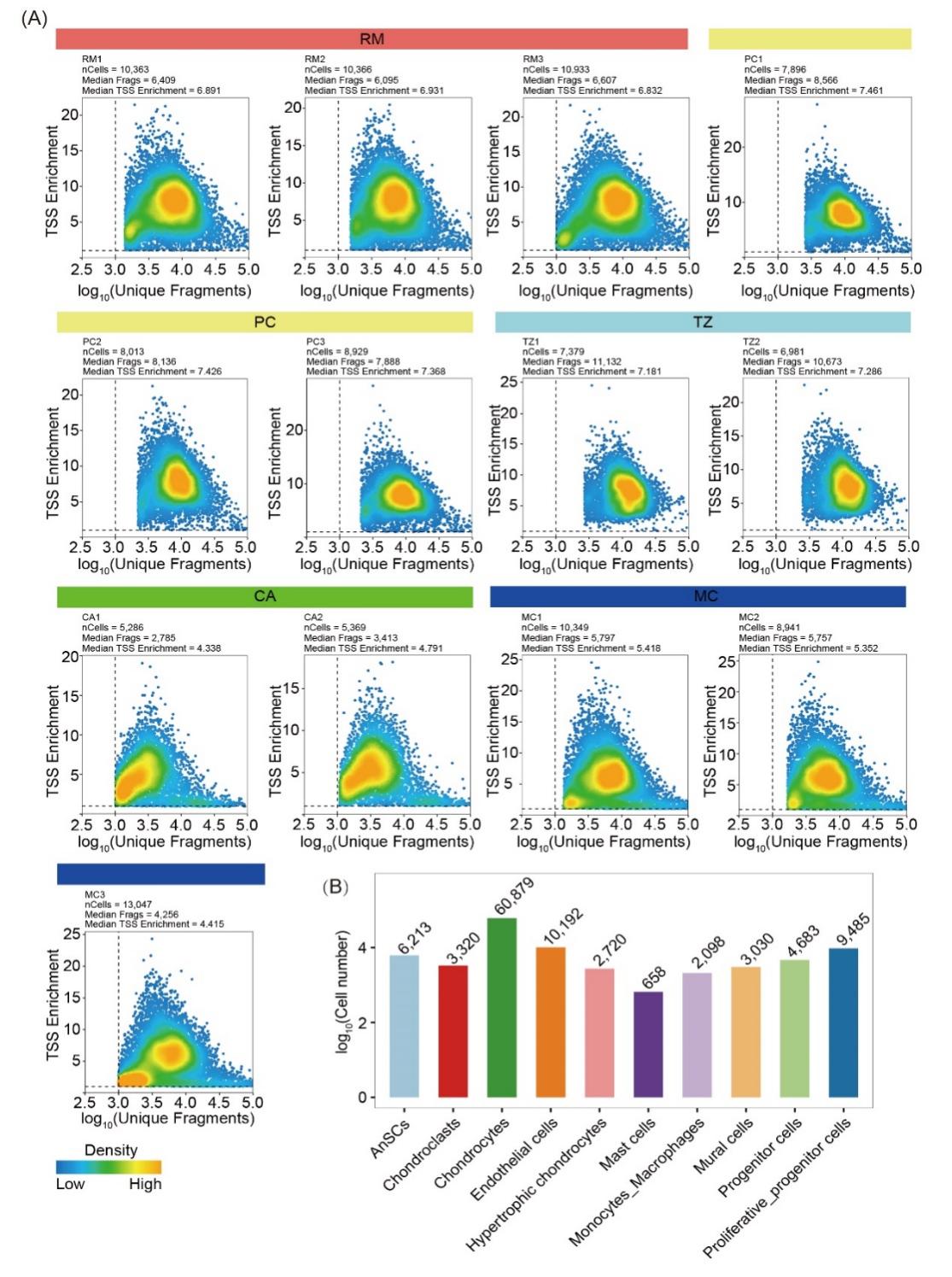
**

**FIGURE S3. Quality control of snATAC-seq data.** (A) Density scatter plot showing transcription start site (TSS) enrichment scores and log-transformed numbers of unique fragments per nucleus. Each dot represents a single nucleus, and color intensity reflects cell density. Dashed lines indicate commonly used QC thresholds (e.g., log_10_[Unique fragments] > 3 and TSS enrichment > 1). High-quality nuclei cluster in the upper-right quadrant and were retained for downstream analysis. (B) Bar plot showing the number of cells retained for each cell type (left) and tissue layer (right) after filtering.

**
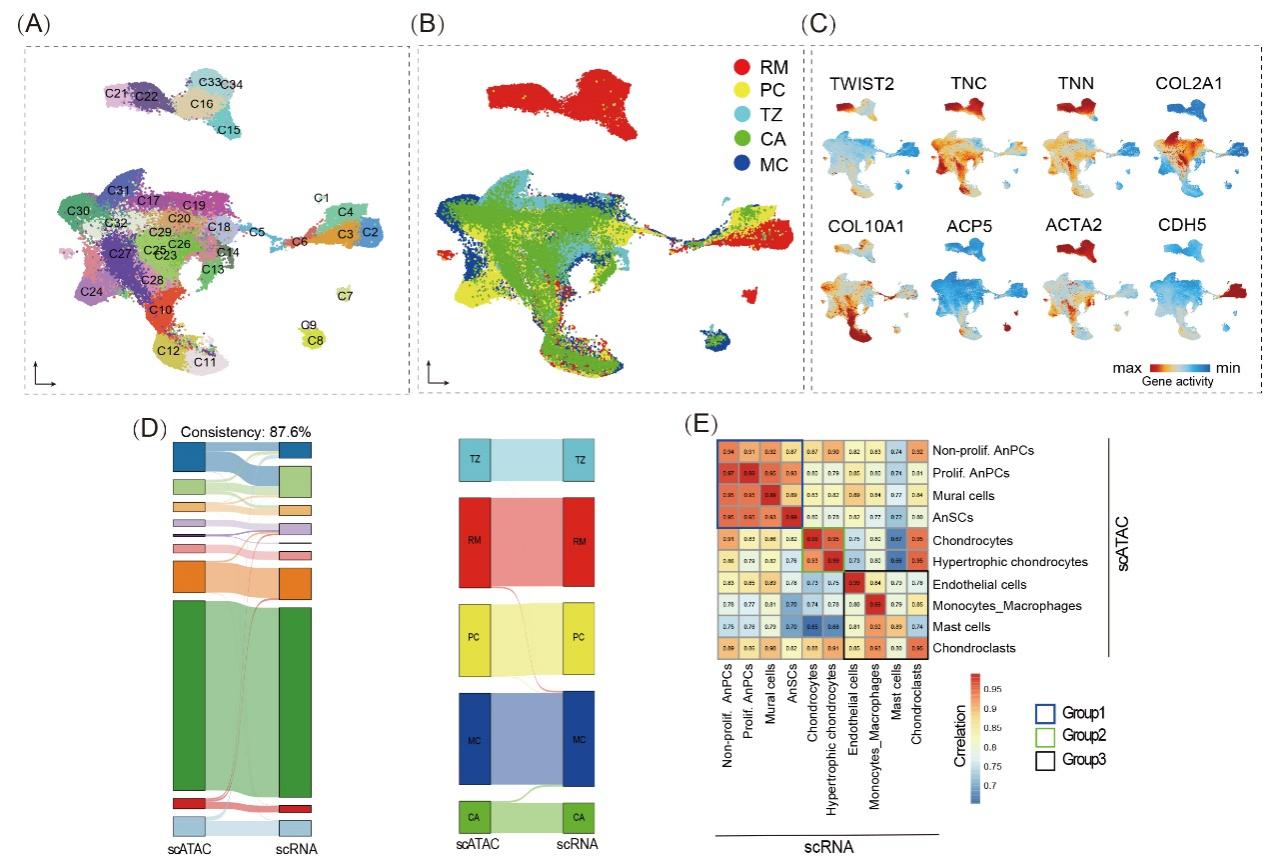
**

**FIGURE S4. snATAC-seq analysis of five tissue layers and integrated multi-omic analysis.** (A) UMAP plot of snATAC-seq data showing unsupervised clustering of all cells from the five layers. (B) UMAP plot showing 10 distinct cell types identified across the five layers, color-coded by tissue layer. (C) UMAP plots showing chromatin accessibility at representative marker gene loci for the 10 cell types. (D) Integrated analysis of snRNA-seq and snATAC-seq data showing concordance at the cell-type (left) and tissue (right) levels. (E) Heatmap of Pearson correlation coefficients between cell types identified by snRNA-seq and snATAC-seq.

**
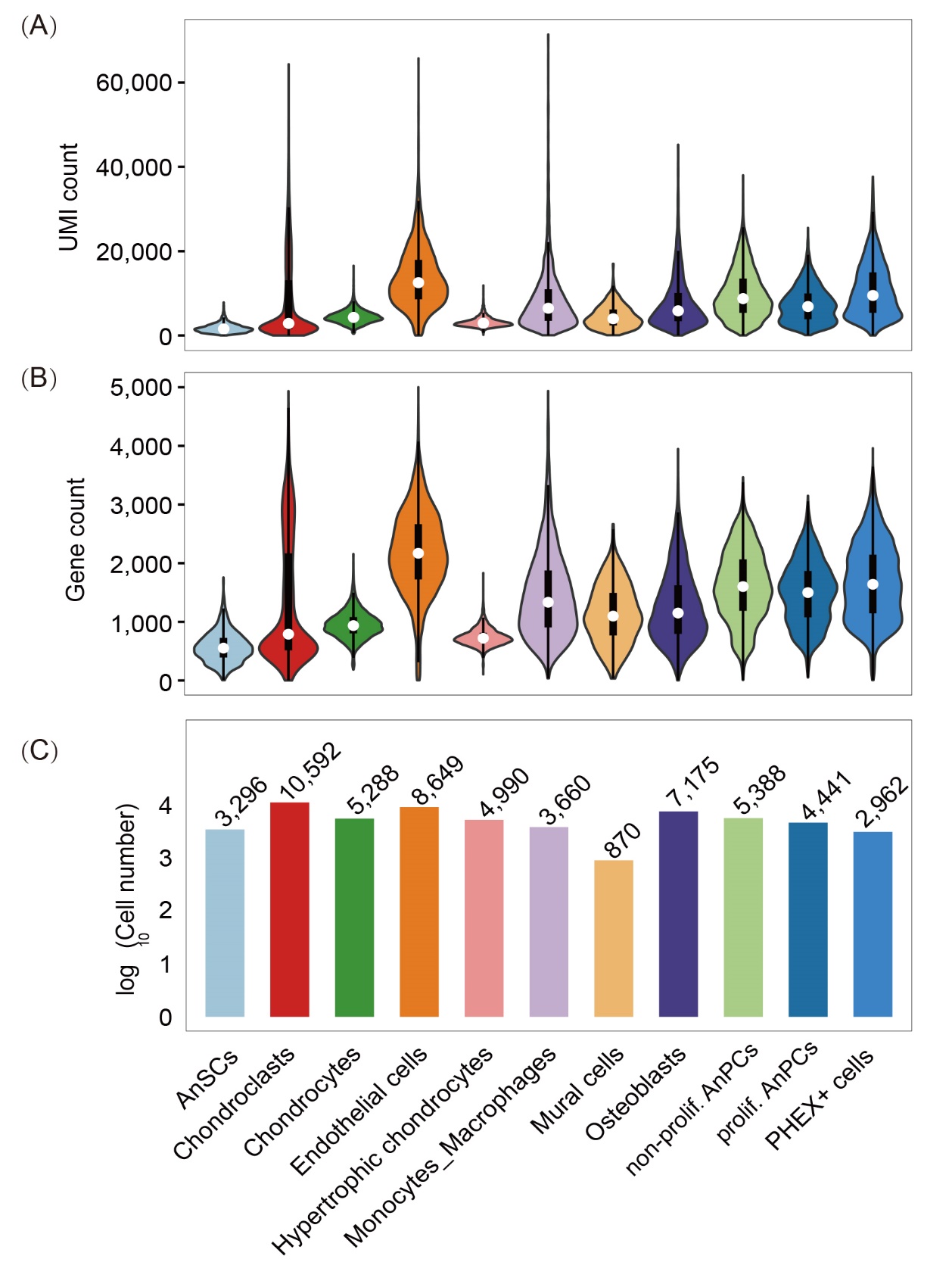
**

**FIGURE S5. Quality control of Stereo-seq data.** (A) Violin plot of UMI counts for each cell type after stringent filtering. (B) Violin plot of detected genes per cell for each cell type after filtering. (C) Bar plot showing the number of retained cells per cell type after filtering.

**
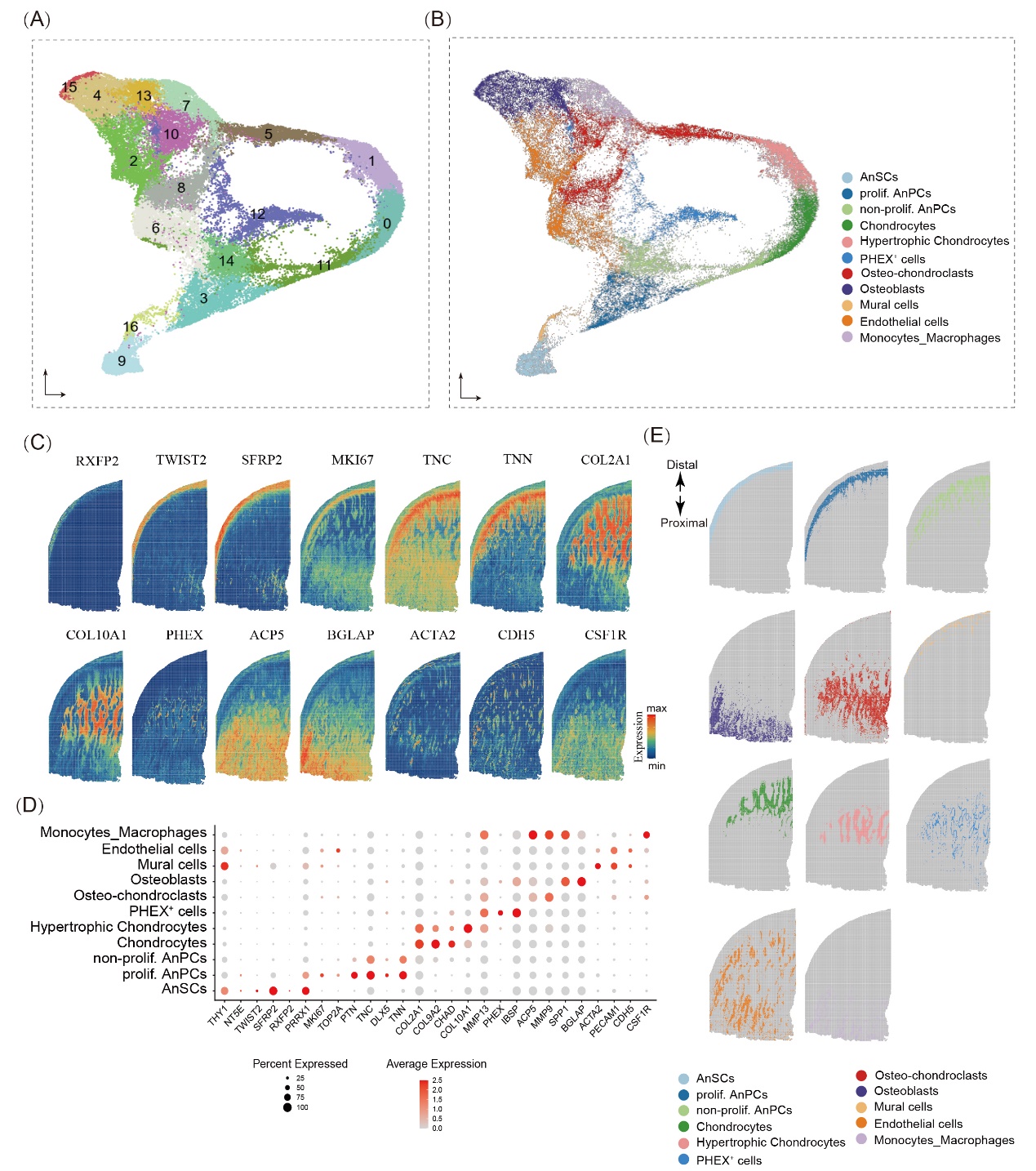
**

**FIGURE S6. Stereo-seq analysis of the AGC.** (A) UMAP plot showing unsupervised clustering of all AGC cells. (B) UMAP plot showing all identified AGC cell types. (C) Spatial expression patterns of representative marker genes for the 11 cell types. (D) Dot plot showing marker-gene expression across cell types. (E) Spatial map visualizing the distribution of the 11 distinct cell populations.

**
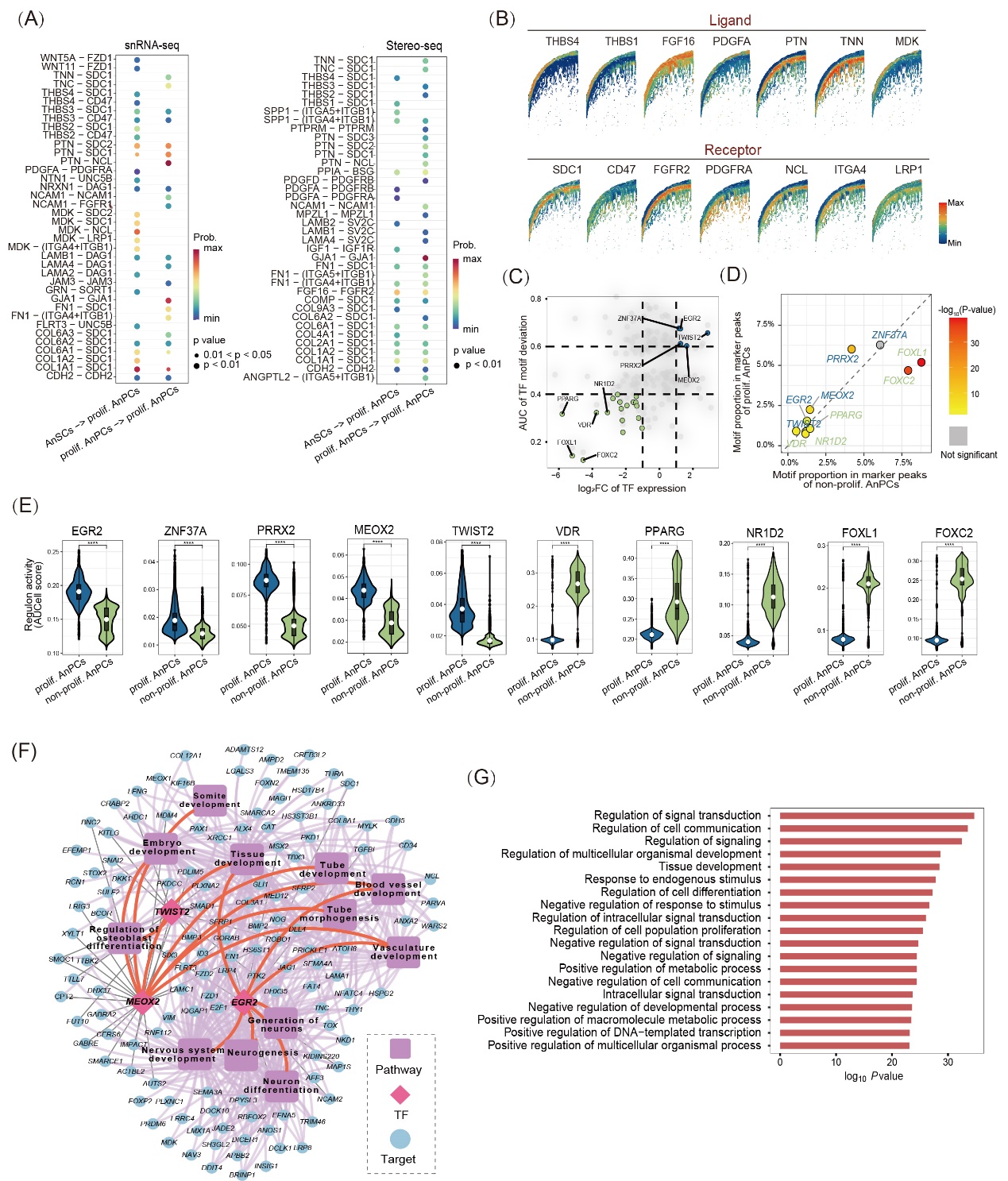
**

**FIGURE S7. AnPC-associated analysis.** (A) Bubble plots showing significant ligand–receptor interactions between AnSCs and proliferative AnPCs, and within AnPCs, based on snRNA-seq (left) and Stereo-seq (right). Key ligand–receptor pairs are highlighted in red. (B) Spatial expression maps of representative ligand–receptor pairs. (C) Scatter plot of activated TFs in proliferative versus non-proliferative AnPCs. Activated TFs were defined as |log_2_FC| ≥ 1 and AUC of motif deviation ≥ 0.6; top five TFs are labeled. (D) Scatter plot showing proportions of differential peaks containing TF-binding motifs in proliferative versus non-proliferative AnPCs. Differences were assessed using the chi-square test. (E) Violin plots of SCENIC regulon activity (AUCell scores) across AnPCs. Top five regulons of proliferative AnPC-activated TFs showed higher activity in proliferative AnPCs, and vice versa. (F) Network visualization of activated TFs in proliferative AnPCs, their predicted targets, and enriched GO biological processes (DAVID), highlighting their multi-lineage differentiation potential. (G) Bar plot of the top 20 enriched DAVID GO biological processes associated with TFs activated in non-proliferative AnPCs and their predicted targets. Note that GO enrichment analysis of non-proliferative AnPCs did not reveal these terms

**
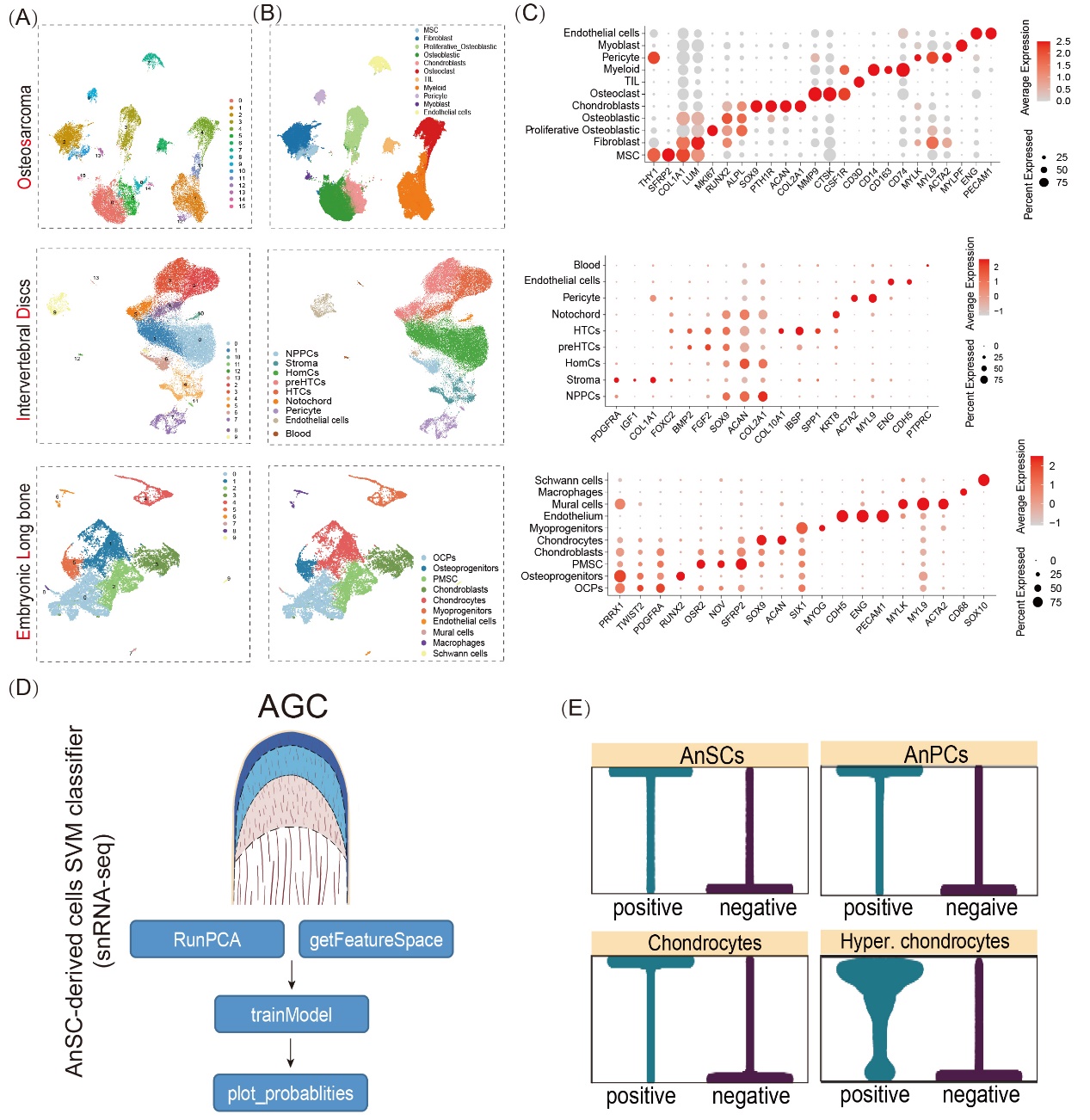
**

**FIGURE S8. Cross-tissue snRNA-seq comparison.** (A) UMAP plots showing unsupervised clustering of cells from osteosarcoma (Os; top), intervertebral disc (ID; middle), and embryonic long bone (EL; bottom). (B) UMAP plots showing identified cell types in ID (top), EL (middle), and Os (bottom). Cell-type abbreviations: NPPCs, nucleus pulposus progenitor cells; HomCs, homeostatic chondrocytes; preHTCs, pre-hypertrophic chondrocytes; HTCs, hypertrophic chondrocytes; PMSCs, perichondrial mesenchymal stromal cells; OCPs, osteo-chondrogenic progenitors; MSCs, mesenchymal stem cells; TILs, tumor-infiltrating lymphocytes. (C) Dot plots showing expression of marker genes across cell types in ID, EL, and Os. (D) Schematic of SVM classifier trained on AnSC-derived cells. (E) Dot plots showing classification probabilities using scPred.

**
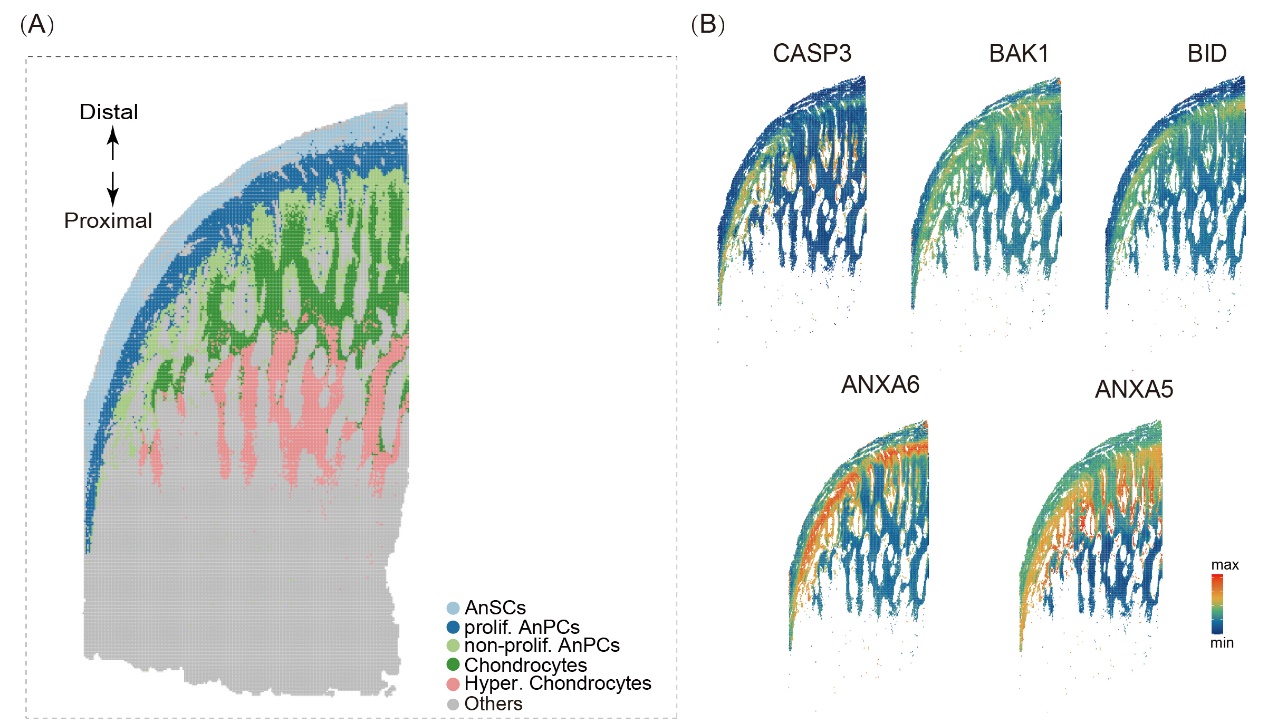
**

**FIGURE S9. Spatial analysis of apoptosis-related genes.** (A) Spatial distribution of AnSCs, proliferative AnPCs, non-proliferative AnPCs, chondrocytes, and hypertrophic chondrocytes. (B) Spatial expression of *CASP3*, *BAK1*, *BID*, *ANXA8*, and *ANXA5*, with predominant expression observed in AnPCs.

**
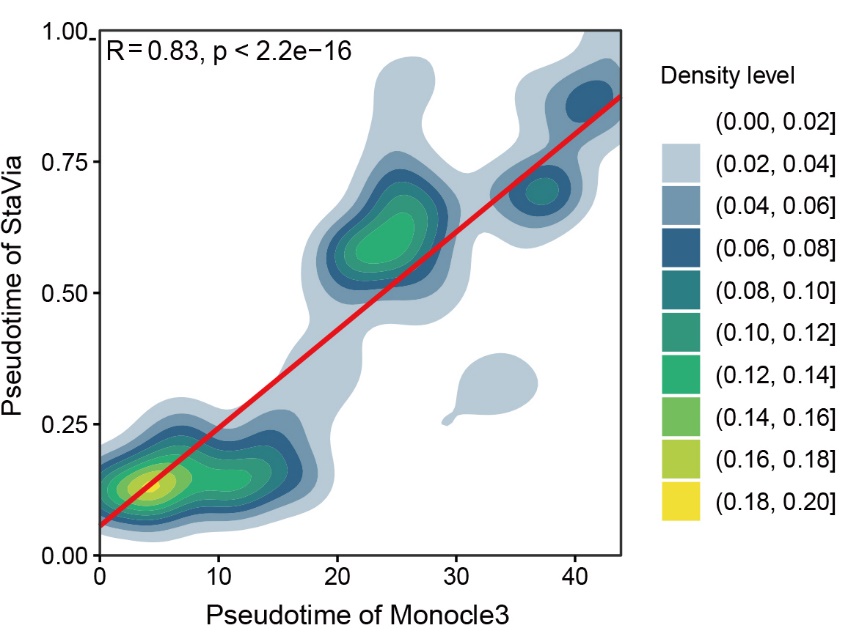
**

**FIGURE S10. Pearson correlation between pseudotime values inferred by StaVia and Monocle3.**

**
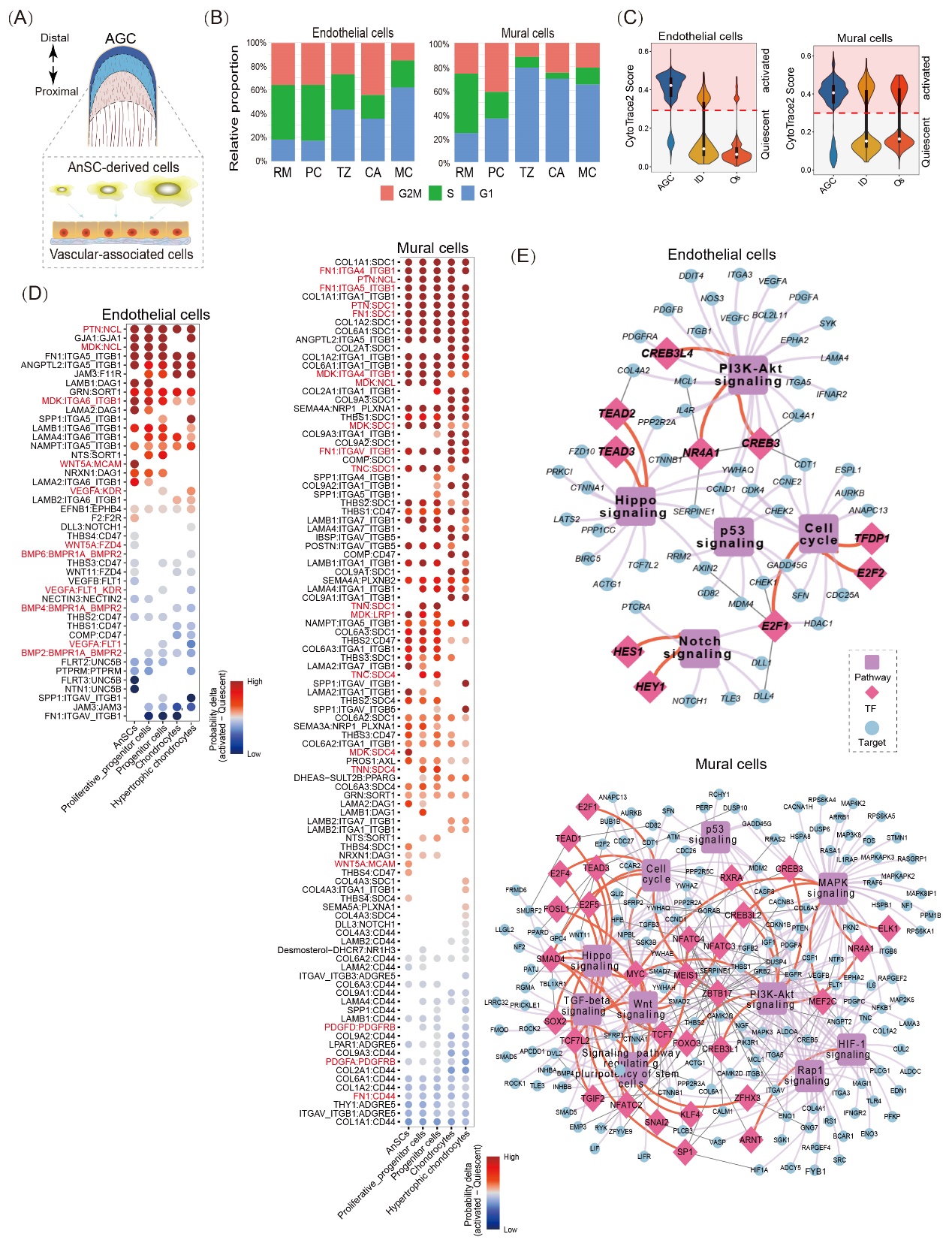
**

**FIGURE S11. Analysis of vascular cells in snRNA-seq and snATAC-seq.** (A) Schematic showing that AnSC-derived cells contribute to vascular microenvironment formation in the AGC. (B) Bar plots of cell-cycle phase composition (G1, S, G2/M) in endothelial and mural cells across the five layers. RM and PC contained more mitotically active vascular cells. (C) Violin plots of CytoTRACE2 scores for endothelial and mural cells across tissues. Cells were classified as activated or quiescent based on a 0.3 threshold. Vascular cells from EL were excluded due to small sample size. (D) Dot plots of differential ligand–receptor interactions (probability delta) between activated and quiescent endothelial (left) and mural cells (right). (E) Network diagrams of activated TFs in activated endothelial (top) and mural (bottom) cells, their predicted targets, and enriched KEGG terms.

**
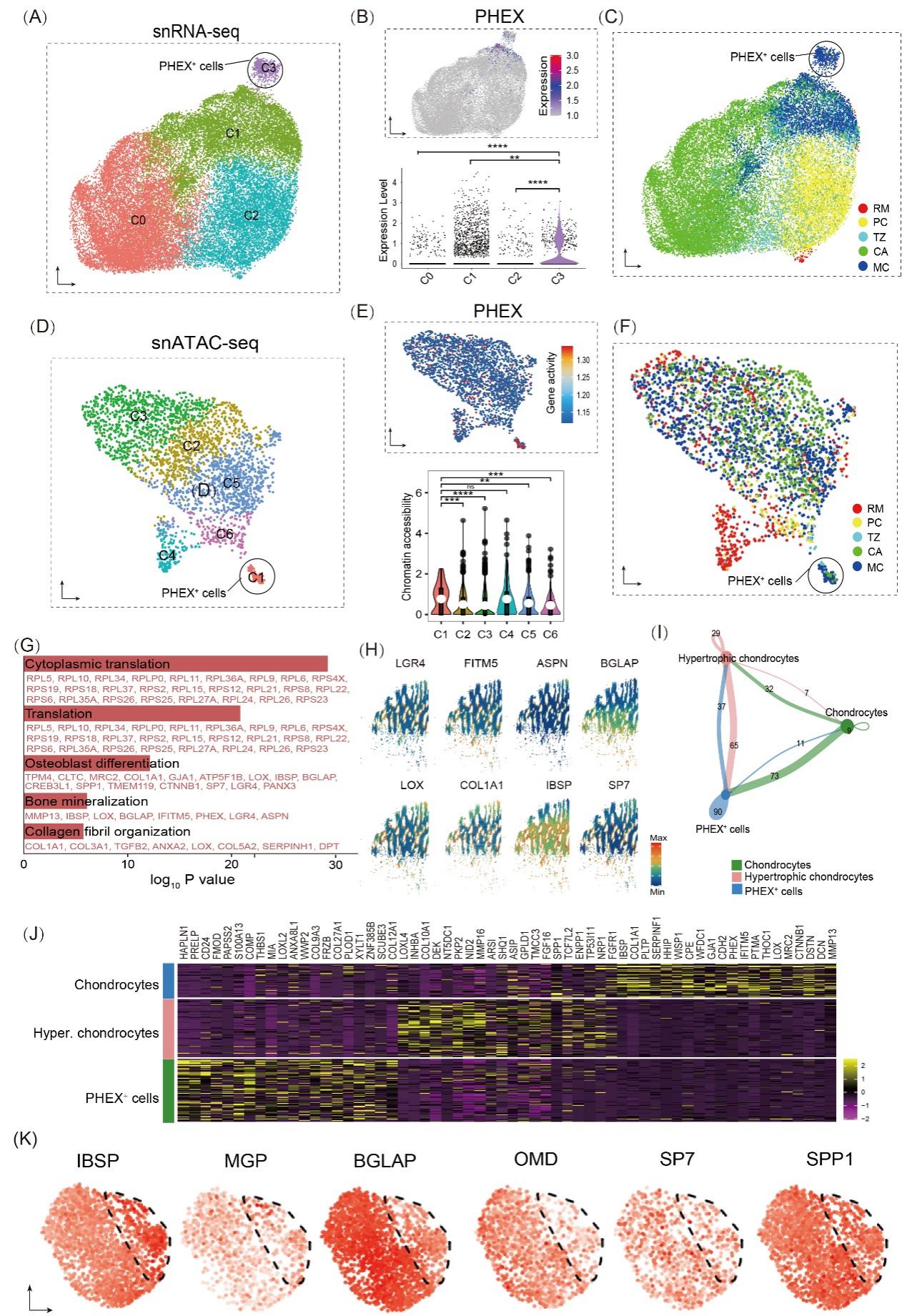
**

**FIGURE S12. Characterization of *PHEX*^+^ cells and hedgehog signaling in chondrocytes.** (A) UMAP plot showing unsupervised clustering of chondrocytes from snRNA-seq. (B) UMAP (top) and violin (bottom) plots showing *PHEX* expression in the snRNA-seq. ***p* < 0.01; *****p* < 0.0001.
(C) UMAP plot showing that *PHEX*^+^ cells predominantly arise from the MC layer in the snRNA-seq. (D) UMAP plot showing unsupervised clustering of chondrocytes from the snATAC-seq. (E) UMAP (top) and violin (bottom) plots showing chromatin accessibility of *PHEX* in the snATAC-seq. ***p* < 0.01; ****p* < 0.001; *****p* < 0.0001. (F) UMAP plot showing that *PHEX*^+^ cells predominantly arise from the MC layer in the snATAC-seq. (G) Bar plot showing the top five DAVID GO biological processes enriched in *PHEX*^+^ cells, including osteoblast differentiation and bone mineralization. (H) Spatial expression of representative bone mineralization genes. (I) Circle plot of predicted cell–cell interactions among chondrocytes, hypertrophic chondrocytes and *PHEX*^+^ cells. (J) Heat map of the top 20 most highly expressed genes in chondrocytes, hypertrophic chondrocytes, and *PHEX*^+^ cells from Stereo-seq. (K) UMAP plot showing expression of osteoblast marker genes.
